# Inhibition of eIF5A hypusination enhances antioxidant defense to prevent kidney Ischemia/Reperfusion injury

**DOI:** 10.1016/j.redox.2025.103814

**Published:** 2025-08-06

**Authors:** Marc Cougnon, Sébastien Giraud, Maria Materozzi, Elisa Allart, Isabelle Rubera, Claire Mackowiak, Gisèle Jarretou, Nadège Boildieu, Virginie Ameteau, Estelle Lemarié, Hajar Ouahmi, Nicolas Melis, Mallorie Poet, Christophe Duranton, Luc Pellerin, Laurent Counillon, Marina Shkreli, Michel Tauc, Thierry Hauet, Didier F. Pisani

**Affiliations:** a Université Côte d’Azur, CNRS, LP2M, France; b Laboratories of Excellence Ion Channel Science and Therapeutics, Nice, France; c Université de Poitiers, INSERM IRMETIST U1313, CHU de Poitiers, Service de Biochimie, Poitiers, France; d Université de Poitiers, INSERM IRMETIST U1313, Poitiers, France

**Keywords:** GC7, Transplantation, Oxidative stress, Catalase, ROS

## Abstract

Ischemia/reperfusion (I/R) refers to the interruption or reduction of blood flow followed by its sudden restoration, resulting in significant oxidative stress, particularly in the kidneys, which are highly oxygen-dependent and metabolically active. During I/R, excessive production of reactive oxygen species (ROS) is triggered by mitochondrial dysfunction and activation of oxidases. Cellular antioxidant defences which attempt to neutralise ROS can become overwhelmed, resulting in oxidative stress that damages macromolecules and ultimately impairs cell function and survival. In kidney transplantation, ROS-induced I/R injury contributes to delayed graft function and chronic graft loss. In this context, inhibition of eIF5A hypusination using the deoxyhypusine synthase inhibitor GC7 protects kidney against I/R injury, potentially by reducing oxidative stress. However, the exact mechanisms and dynamics of this antioxidant protection remain to be elucidated.

Using a mouse model of renal I/R and equivalent *in vitro* cell model, we analyzed the concomitance between protection against oxidative stress due to GC7 treatment and recovery of renal function or cell survival. In addition, we analyzed proteome modulation due to GC7 treatment to unravel pathways involved in its protective effect, and we defined the impact of GC7 on ROS productions and on antioxidant defences.

We demonstrated that GC7 protected against I/R-induced injury and anoxia/reoxygenation in both *in vivo* and *in vitro* models by conditioning the cells and organ to resist stress. From a mechanistic point of view, we showed that the protective effects of GC7 were largely attributed to the enhancement of antioxidant defences, mainly through sustained catalase activity, which was mandatory in kidney cells to survive in the face of ROS production.

Overall, GC7 is a clinical candidate for reducing oxidative damage in kidney transplantation, particularly for organs from marginal donors. Its ability to reprogram redox and metabolic pathways early after treatment supports its use to improve graft survival and function.

## Highlights

• GC7 improves recovery of renal function after ischemia/reperfusion in mouse.
• GC7 protects the kidney against oxidative stress during ischemia/reperfusion.
• GC7 treatment modifies the kidney cell proteome rapidly and maintains it over time.
• GC7 increases antioxidant defense of kidney cells *in vivo* and *in vitro*.
• Catalase activity is crucial for kidney cell protection against oxygen deprivation.

## 1 Introduction

Ischemia/reperfusion (I/R) is described as the process covering blood flow arrest or its reduction (low flow), causing oxygen and nutrients deprivation, and the blood reflow, leading to a sudden reoxygenation [1,2]. I/R can occur suddenly and unexpectedly in pathological situations (stroke, infarct …) but also in controlled settings such as arterial clamping during organ procurement and transplantation, and its known to induce irremediable lesions [1]. Among organs and tissues, kidneys are extremely sensitive to ischemia: they account for 10 % of the whole-body O_2_ consumption and are very active metabolically [3]. Kidney dependence to O_2_ is exacerbated compared to other organs due to a renal precapillary oxygen shunting between arterial and venous vessels. Notoriously, this leads to a faster decrease in cortical microvascular O_2_ pressure when blood oxygenation decreases [[4], [5], [6]]. Conversely, this arterial-venous shunt grants kidneys a mild protection towards reperfusion by slowing down the rise in O_2_ pressure [7].

These changes in O_2_ availability during I/R, associated with IR-induced mitochondrial damage, drive an excessive and unbalanced production of reactive oxygen species (ROS) [8]. In absence of effective regulation by the cell antioxidant defenses, ROS can react with and disrupt macromolecules, causing fast lesions of cells and tissues, expanding damaged areas [9,10]. During kidney transplantation, ROS-induced I/R injury is the most critical as they significantly raise the risk of delayed graft function, prime the donor kidney for rejection, and globally contribute to the long-term risk of graft loss. Thus, limiting I/R lesions due to ROS is paramount for efficient treatments [11,12].

ROS are unstable oxygen derivatives (superoxide anion (O_2_•-), hydroxyl radical (HO•) …), highly reactive with surrounding molecules and produced through various processes, such as oxidases activity and mitochondrial respiratory chain [13]. Combination of ischemia-associated functional alterations of complexes I and III and sudden reoxygenation along with substrate availability drive O_2_•- production [[14], [15], [16], [17], [18]]. Among other ROS sources during I/R, Xanthine Oxidoreductase complex can generate vast amount of ROS specifically during reperfusion [8]. Indeed, during ischemia, purine catabolism increases and causes accumulation of hypoxanthine into the cell. At reperfusion, high quantities of hypoxanthine are oxidized to xanthine by Xanthine Dehydrogenase (XDH) which in turn is oxidized to uric acid by Xanthine Oxidase (XO, converted form of XDH) concomitantly to the production of O_2_•- [19]. Finally, the NADPH oxidase family (NOX and DUOX) is also involved in the production of extracellular O_2_•- by reduction of one electron of oxygen using cytoplasmic NADPH as an electron donor [20]. O_2_•- is mainly detoxified by mitochondrial (Mn-SOD) and cytoplasmic (Cu/Zn-SOD) superoxide dismutase (SOD) into hydrogen peroxide (H_2_O_2_), a more stable oxygen derivative. H_2_O_2_ is then converted into H_2_O either by the catalase (CAT), the peroxidase domain of DUOX or by the glutathione peroxidase (GPx) [21]. SODs, CAT, and peroxidases are the main component of the cell antioxidant defence. When CAT and peroxidases are overrun, H_2_O_2_ can then react with transition metals, such as Iron (Fe) and Copper (Cu) via the Fenton reaction, to generate the highly reactive HO• [9,12].

Importantly, at physiological concentrations, ROS are necessary to fulfil various cell functions, and their levels are equilibrated between their generation and the antioxidant systems [21]. Overproduction of ROS or a diminished antioxidant potential lead to oxidative stress causing irreversible oxidation of biological macromolecules (DNA, proteins and lipids), altering cell function and ultimately causing cell death [22]. Moreover, Reactive Nitrogen Species (RNS), and particularly Peroxynitrite (ONOO•-), generated by excessive O_2_•- reacting with Nitric Oxide (NO) produced by iNOS (NOS2), participate to the harmful oxidative stress, particularly during reperfusion and have similar nefarious effects on tissues, initiating lipid peroxidation as well as protein nitro-tyrosination, two highly deleterious cytotoxic processes involved in kidney transplantation injuries [23,24].

We have previously demonstrated that hypusination inhibition of eukaryotic translation Initiation Factor 5A (eIF5A) was an effective anti-ischemic strategy in kidney transplantation [25,26]. Hypusination, a post-translational modification of eIF5A, is described as the addition of a moiety from the polyamine spermidine, on a lysine residue of eIF5A via the successive enzymatic activity of deoxyhypusine synthase (DHPS) and deoxyhypusine hydroxylase (DOHH). Hypusination of eIF5A is unique, extremely conserved in living organisms, and seems to control the translation of a specific subset of proteins mainly involved in oxidative functions [[27], [28], [29]]. In a rat model of I/R through renal artery clamping, we demonstrated that the inhibition of eIF5A hypusination by the spermidine analogue and specific DHPS inhibitor GC7 (N1-guanyl-1,7-diamineoheptane) protected renal function from I/R injury [26]. We then demonstrated GC7 preconditioning protective effect in pre-clinical models of kidney transplantation with early graft functional recovery and prevention of late interstitial fibrosis development [25,26]. In these studies, we suggested a protective effect of GC7 treatment against oxidative stress induced by I/R, as it decreased ROS production in mouse proximal convoluted tubule cells *in vitro*, as well as *in vivo* in pig in which a strong reduction of Nitro-tyrosine protein level was observed [25,26]. This could partially explain the decrease in fibrosis, as early oxidative stress has been linked to development of a fibrogenic environment [30]. Nevertheless, these results did not evidence a direct correlation between anti-ischemic and antioxidant effects. Moreover, they did not uncover the mechanisms allowing protection against oxidative stress injury during I/R in GC7-treated cells and organs, as well as the dynamic of this protection.

In the present study, by an approach using both an *in vivo* model of murine renal I/R and an *in vitro* model of proximal tubule cells, highly sensitive to I/R, we evidence that 1/GC7 protects renal tissue and cells from early oxidative stress post-I/R, positively correlated with a better functional recovery of the kidney, preventing cell death and that 2/GC7 operates via the modulation of a network of proteins involved in redox homeostasis, promoting antioxidant defenses rather than limiting ROS production during anoxic stress. Taken together, those results show, for the first time, a direct link between hypusination inhibition and oxidative stress and prevention of kidney I/R associated oxidative stress lesions.

## 2 Material & methods

**Reagents.** When not specified, drugs, buffer solutions, fetal bovine serum (FBS) and culture reagents were from Sigma-Aldrich Chimie (*Saint-Quentin-Fallavier, France*). N-guanyl-1,7-diaminoheptane (GC7) was synthesized by AtlanChim Pharma (*Nantes, France*) according to the previously described method [31].

**Animal Model.** An established mouse model of unilateral renal ischemia-reperfusion was used [32]. C57BL6/J male mice, 10 weeks old, from Charles River Laboratory (*Ecully, France*) were injected intraperitoneally with GC7 (3 mg/kg) or vehicle (NaCl 0.9 %) and subjected 4h later to left nephrectomy and right renal IR (IR-group) or to the same surgical procedure without nephrectomy and renal clamp (Sham group). Briefly, mice were anesthetized with isoflurane (4 % for induction and 1.5 % for maintenance). Under anaesthesia and analgesia, after flank incision, the left contralateral kidney was ligated, and the right renal pedicle was clamped using a straight Schwartz Micro clip (Fine Science Tools, *Heidelberg, Germany*) for 32 min and then released for reperfusion. Blood and kidneys were collected at indicated hours from 0 (H0) to 3 (H3) hours after reperfusion or days from day −7 to day 30 (D30) after reperfusion. Control (CTRL) values were obtained from normal C57BL6/J Mice 10 weeks old (kidneys and blood). All animal experimental procedures and housing were carried out in accordance with the European Communities Council Directive (2010/63/EU), ARRIVE guidelines, and complied with the three Rs principle (Replace, Reduce, Refine). The animal study was reviewed and approved by a regional Ethics Committee (COMETHEA Poitou-Charentes, authorization n°39546–2022110817149989).

**Cell Culture.** Renal proximal convoluted tubule cells (PCT) were obtained from primary cultures of murine proximal tubule segments, immortalized with pSV3neo vector and were cultured as previously described [33,34]. All experiments were performed the day after cell confluence.

*Drug treatments.* PCT cells are treated with 30 μM GC7 for 8 h and used directly after treatment for proteomic analysis, or washed and used after an additional overnight culture (+16 h) for all others experiments. The different inhibitors are used as pretreatment before stress procedure: buthionine sulphoximine (BSO) 100 μM, 24 h; 3-amino-1,2,4-triazole (3-AT) 30 mM, 30 min or 1 h; pyocyanin 20 μM, 1 h; AgNO_3_ (Silver Nitrate) 5 μM, 1 h.

*Stress conditions.* Mitotoxic treatment consisted of submitting the cells with 1 μM myxothiazol or 5 μM antimycin A for 24 h. H_2_O_2_ treatment (10 mM) was performed at 37 °C for 20 min. For anoxia, cells were maintained 4 h at 37 °C in an airtight chamber containing a 100 % N_2_ atmosphere. Oxygen deprivation was controlled using an OXYBABY® apparatus (WITT, *Morsang sur Orge, France*). Reoxygenation mimicking reperfusion was performed by placing cells for 2 h under 5 % CO_2_/95 % air water-saturated atmosphere at 37 °C.

**Proteomic processing and analysis.** PCT cells were lysed with a RapiGest buffer (Waters, *Massachusetts, USA*). Lysates were sonicated using a Vibra Cell Sonicator (VWR, *Pennsylvania, USA*) and diluted in 8 M urea in 1 M pH 8 Tris-HCl followed FASP digestion using a 10 kDa filter (Merck Millpore, *Massachusetts, USA*) to perform successive reduction (dithiothreitol), alkylation (iodoacetamide) and incubation with trypsin/LysC (Promega, *Charbonnières-les-Bains, France*). Protein digests have been analyzed using an Acclaim PepMap C18 column (75 μm × 15 cm, 2 μm) reverse phase chromatography (ThermoFisher Scientific) with a binary gradient of formic acid (0.1 % v/v in water and 0.1 % v/v in acetonitrile). The eluent outlet was connected to the MS analyzers for detection and characterization. A nano electrospray ion source and Orbitrap mass spectrometer for measurements of accurate mass have been conducted with a QExactive instrument (ThermoFisher Scientific). Data were acquired in positive mode by using the Orbitrap at high mass resolution for scans. The peptide MS/MS data were recorded using a data-dependent acquisition method (DDA) scanning approach with the selection and fragmentation of multicharged ions.

The data output was normalized and processed for protein identification against the mouse Uniprot protein database. Semi-quantitative analysis of proteome has been performed using a Progenesis ion abundance method based on a topmost abundant peptide quantification. The data search was performed by the Proteome Discoverer 2.3 software coupled with SEQUEST search engine (ThermoFisher Scientific) for peptide identification using a false discovery rate (FDR) < 1 % set for both peptide and protein identification. Relative quantification of identified proteins was made by Progenesis 4.1 (Waters®). Quantified intensities were imported in Perseus v2.0.11, transformed in log2, filtered for genes identified only in small number of samples and imputed through normal distribution (width 0.3; down shift 1.8) resulting in a list of 3183 proteins (full list with imputed values is available in Supplementary Data 1). Statistically significant proteins (Student T test p < 0.05) were selected for gene pathway enrichment with ClueGO v2.5.10 (Cytoscape v3.10.3) in the databases of KEGG_May 25, 2022, GO_MolecularFunction-EBI-UniProt-GOA-ACAP-ARAP_May 25, 2022, GO_CellularComponent-EBI-UniProt-GOA-ACAP-ARAP_May 25, 2022, GO_BiologicalProcess-EBI-UniProt-GOA-ACAP-ARAP_May 25, 2022, with the following parameters: min_associated genes = 5 %; Kappa_score_threshold = 0.4; min_GO_level = 3; Max_GO_level = 8; min_genes = 5; p-value cutoff = true, Correction_method = Benjamini-Hochberg. Representative graphs were made with GraphPad Prism v10 (full list of pathways and associated genes in Supplementary Data 2 and 3). Network analysis was performed with STRING v12.0, identified GO are specified in the legend of each network. Heatmap representation was generated with Morpheus (https://software.broadinstitute.org/morpheus).

**Cell survival analysis.** Cell viability was evaluated by using Calcein-AM 1 μM (#C3099, Invitrogen™ (ThermoFisher Scientific, *Courtaboeuf, France*)) and Ethidium homodimer 4 μM (#E1903, Sigma-Aldrich) to stain live and dead cells respectively. Cells were incubated for 30 min at 37 °C in presence of both dyes and then washed twice with HBSS (w/o Phenol Red) before analysis. Fluorescence signals were recorded using an observer D1 microscope (Carl Zeiss, *Le Pecq, France*) and analyzed with Fiji software using homemade macro [35]. Results are expressed as “survival rate” compared to control normoxia condition.

**Immunoblotting.** Whole proteins from cells or kidney cortex were prepared using TNET lysis buffer (25 mM Tris-Cl (pH 7.4), 100 mM NaCl, 1 mM EDTA, 1 % w/v Triton X-100, 0.5 % w/v Nonidet P40, 1x protease and phosphatase inhibitor cocktail (Sigma-Aldrich)). Tissues were disrupted using Precellys apparatus (Bertin Technologies, *Montigny le Bretonneux, France*) and microbeads (Ozyme, *Saint Cyr* L*’école, France*). Crude lysate was centrifuged at 10000 g (30 min, 4 °C). Supernatants containing soluble proteins were preserved for analysis. Protein concentration was evaluated using DC reagent assay (BioRad, *Marnes-la-Coquette, France*) and blotted using SDS-PAGE basic protocol and Mini-PROTEAN® TGX™ Precast Protein Gels (BioRad). Primary antibody incubation was performed overnight at 4 °C (1:1000, bovine serum albumin 3 % w/v; anti-SARS1 (#ab154825), anti-SOD1 (#ab51254), anti-SOD2 (#ab13533) and anti-eIF5A (#ab137561) are from abcam (*Amsterdam, Netherlands*), anti-p62/SQSTM1 (#P0067) and anti-β-actin (#A5441) from Sigma-Aldrich, anti-β-tubulin (#MAB16308) from ThermoFisher Scientific, anti-catalase (#14097) from Cell Signaling technology (Ozyme), and anti-hypusine (#PTX18841) from Proteogenix (*Schiltigheim, France*)) and then blots were incubated at RT 30 min with an adequate HRP-conjugated secondary antibody (bovine serum albumin 3 % w/v, 1:5000, Jackson ImmunoResearch, *Ely, United Kingdom*). Detection was performed using chemiluminescent HRP substrate (Amersham, *Freiburg, Germany*) and Fusion FX imaging system (VILBER, *Marne-la-Vallée, France*). Band intensities were evaluated using Fiji Software [35]. Uncropped Western blots are available in Supplementary Fig. 4.

**Histology, Immunohistochemistry.** Kidney splits were fixed with 4 % formalin and paraffin embedded. Embedded tissues were cut in 5 μm sections and dried 30 min at 55 °C. Sections were then deparaffinized in xylene, rehydrated through alcohol, and washed in phosphate-buffered saline (PBS).

Evaluation of renal interstitial fibrosis was performed by Sirius Red staining.

For immunohistochemistry/fluorescence, antigen retrieval was performed using Vector unmasking reagent (#H3300, Vector Laboratories). Vimentin expression (marker of epithelial-mesenchymal transition) was assessed by immunohistochemistry (#347M − 16, Sigma-Aldrich). Rabbit antibodies targeting HADH (#19828-1-AP, Proteintech) and SARS1 (#ab154825, AbCam) were detected using an AlexaFluor488-conjugated goat anti-rabbit secondary antibody (1:300) (#111-545-144, Jackson ImmunoResearch).

Acquisitions were performed on brightfield and confocal microscopy for immunohistochemistry and immunohistofluorescence respectively. Staining quantifications were performed using Fiji software. All evaluations were performed and scored under blinded conditions.

### 2.1 Oxidative stress analysis

*In vitro analysis*. Cells were grown in 96 wells black plate with clear bottom (PerkinElmer, *Villebon-Sur-Yvett, France*). For anoxia/reoxygenation experiments, cells were incubated prior to stress in HBSS containing 1 % w/v FBS either with 5 μM MitoSox Red (396/610 nm, #M36008, Invitrogen™) for measurement of mitochondrial superoxide anion levels; with 2 μM MitoTracker Green FM (490/520 nm, #M7514, Invitrogen™) for determination of mitochondrial mass to normalized MitoSox signal; with 5 μM CM-H2DCFDA (490/520 nm, #C6827, Invitrogen™), a probe mainly sensitive to hydroxyl radical and peroxynitrite, or with 2 μM Hoechst 33342 (360/490 nm, #62249, Invitrogen™) to stain nuclei of live cells to normalize CM-H2DCFDA signal. Fluorescence intensity was determined every 5 min for 2 h at 37 °C using a SP2000 Xenius plate reader immediately after anoxia (SAFAS, *Monaco*). Signals of the 4 probes were recorded sequentially at each time point. For H_2_O_2_ stress, the same protocol was used except that cells were incubated 30 min with H_2_O_2_, washed 3 times with HBSS and then incubated with probes before being immediately analyzed for fluorescence intensity for 2 h.

*In vivo analysis*. The Amplex® Red reagent, in combination with reaction buffer and horseradish peroxidase (HRP), has been used to detect H_2_O_2_, on OCT renal tissue sections with the Amplex Red (Hydrogen Peroxide Assay Kit, #A22188, Invitrogen™). After incubation and washing, sections were mounted using a mounting medium containing DAPI (#ab104139, Abcam). Quantifications were performed and scored by a blind experimenter using Fiji software.

### 2.2 Others biochemical analysis

All fluorescence and absorbance were determined using a SP2000 Xenius plate reader (SAFAS, *Monaco*).

*Cell enzymatic activity.* GSH cell contents were evaluated as previously described [36]. NO content (#MAK454), lipid peroxidation (#MAK085) and protein carbonylation (#MAK094) assays were from Sigma-Aldrich and performed following manufacturer's instructions. Catalase activity was measured using colorimetric assay (#MAK381 from Sigma-Aldrich and #15860893 from Invitrogen).

*Tissue enzymatic Activity*. Amplex® Red reagent, in combination with H_2_O_2_, has been used to measure the peroxidase activity (that enables the hydrogen peroxide (H_2_O_2_) to be converted to H_2_O) in renal tissue lysate samples with the Amplex Red Hydrogen Peroxide Assay Kit, according to the manufacturer's instructions (#A22188, Invitrogen™).

Superoxide dismutase (SOD) activity (including Cu/Zn-, Mn- and Fe-SODs) was measured in renal tissue lysate samples with the superoxide dismutase assay kit (#706002, Cayman Chemical, *Ann Arbor, Michigan, USA*), which utilizes a tetrazolium salt for a detection of superoxide radicals generated by xanthine oxidase and hypoxanthine. according to the manufacturer's instructions.

Catalase activity was measured in renal tissue lysate samples with the Catalase Colorimetric Activity Kit (#EIACATC, Invitrogen™). Samples were incubated 30 min with hydrogen peroxide reagent, then horseradish peroxidase and substrate were incubated 15 min.

*Blood parameters.* Biochemical analyses, on plasma obtained from blood samples, were performed at the Biochemistry department of the Poitiers Hospital on an automated Roche Cobas® system (Roche Diagnostics, *Meylan, France*) for Urea nitrogen (BUN, in mmol/L) and Creatinine (μmol/L) measurement. Malondialdehyde (MDA in μmol/L) measurement were performed with a Colorimetric Assay Kit (#EEA015, Invitrogen™), on plasma obtained from blood samples, according to the manufacturer's instructions.

**Statistical analysis.** Results were analyzed by GraphPad Prism 10 software. Statistical differences between experimental groups were defined as indicated in figure legends.

## 3 Results

### 3.1 Correlation between kidney function recovery and oxidative stress after renal I/R

**Fig. 1:**
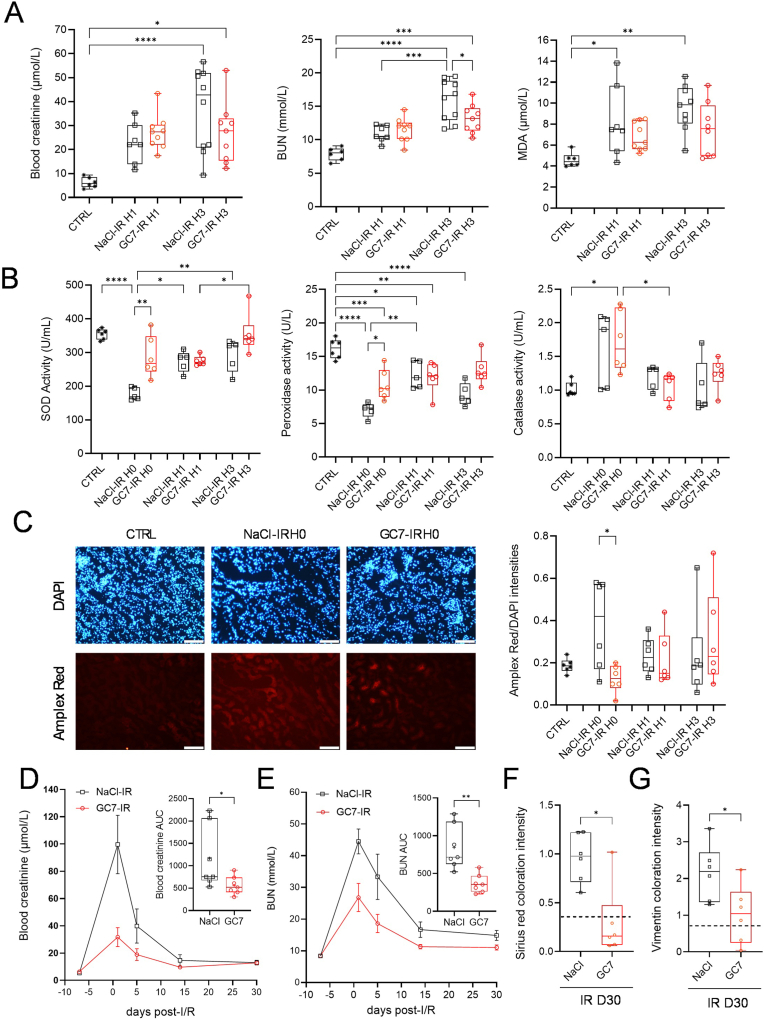
**Kidney injuries and function recovery after renal Ischemia/Reperfusion in mice treated with GC7.** Mice were injected intraperitoneally with GC7 (3 mg/kg) or vehicle (NaCl) and subjected 4h later to a left nephrectomy and a 32 min right renal ischemia followed by reperfusion. Mice were then followed from 0h to 3h (A–C) or at 30 days (D–G) post-reperfusion. (A) Blood creatinine, blood urea (BUN) and blood MDA were evaluated at indicated hours with H0 = end of ischemia. (B) At the same time points, Catalase, SOD and peroxidase activities were evaluated in whole kidney lysates. (C) Oxidative stress was evaluated by Amplex Red reagent on kidney slices at indicated hours. Amplex Red signal was normalized by the intensity of DAPI staining. (D) Blood creatinine and (E) BUN were evaluated at the indicated time, with day 0 = I/R. Curves represent means ± SEM, n = 7 and the corresponding area under the curve (AUC) calculated for each mouse is shown and displays individual values and means ± SD. (F–G) Histology analysis of kidney sections at day 30 after I/R (IR D30). (F) Quantification of Sirius red staining visualized by normal light to evaluate collagen I and III fibers and (G) of vimentin immunodetection. Boxes displayed individual values and their distribution, mean, minimum and maximum. When displayed, dotted line represents mean obtained from 6 normal mouse kidneys. ∗p < 0.05, ∗∗p < 0.01, ∗∗∗p < 0.001, ∗∗∗∗p < 0.0001, Kruskal Wallis & Dunn's multiple comparison test or Mann-Whitney's comparison test for 2 groups.

To elucidate the potential antioxidant effects of GC7 during I/R and the subsequent effect on kidney function recovery, we chose to evaluate its potential in a murine model of uninephrectomy-unilateral renal I/R. Animals were treated with GC7 at 3 mg/kg (GC7-IR) or its vehicle only (NaCl-IR) 4h prior to a left nephrectomy procedure concomitant to a right kidney artery clamping for 32 min and a monitoring for either a few hours, to evaluate early events, or after 30 days for late injury. Blood creatinine as well as urea nitrogen (BUN) slightly increase independently of treatment at 1-h post-reperfusion (H1) and more at 3-h (H3) (Fig. 1A), indicative of the expected kidney lesion due to I/R with contralateral nephrectomy. However, GC7 treatment partially prevented the high blood creatinine and BUN levels observed upon IR in the NaCl group when compared to CTRL. Indeed, while the NaCl-IR group exhibited a marked increase of blood creatinine at 3 h post-reperfusion, the GC7-IR group showed a less significant increase at the same time point (Fig. 1A). Importantly, BUN values were significantly reduced at 3 h post-reperfusion after GC7 treatment compared with the NaCl-IR group (Fig. 1A). In addition, blood levels of MDA (Malondialdehyde, a marker of oxidative stress) increased significantly only in NaCl-treated mice at both 1 and 3 h after reperfusion (Fig. 1A) suggesting a lower oxidative stress in GC7 treated mice.

To confirm this, we analyzed the antioxidant enzymatic activity at the end of ischemia (H0) and during the early phase of reperfusion (H1–H3): while superoxide dismutase (SOD) and peroxidase activities dropped dramatically at the end of ischemia (H0) in the vehicle group, GC7 pretreatment prevented this decrease (Fig. 1B). During the 3 h following reperfusion, antioxidant enzymatic activity slowly increased to reach pre-ischemia values for SOD and peroxidase (Fig. 1B). Catalase activities were too heterogenous in the vehicle NaCl group to be conclusive: some mice displaying increased activity after I/R, while others showed no modulation (Fig. 1B). On the contrary, GC7-treated mice displayed an homogeneous and significant increase in catalase activity at the reperfusion (H0) and then returned to basal values (Fig. 1B). This demonstrated that GC7 could maintain and/or increase the renal antioxidant activity during I/R and that the antioxidant cascade is reinforced by GC7, as particularly demonstrated by the increased catalase activity. This was validated by an increased H_2_O_2_ (Amplex red staining on kidney sections) in vehicle-treated mice at the end of ischemia, but not in GC7-treated mice, suggesting efficient transformation of H_2_O_2_ in H_2_O by catalase (Fig. 1C).

We then evaluated the protective effect of GC7 on renal function recovery and late injury. GC7 significantly prevented the increase in blood levels of creatinine and urea (Fig. 1D and E respectively), characteristic of renal function failure. Specifically, these parameters are significantly increased at day 1 and 5 post-I/R in the vehicle group while in the GC7 group, only a slight increase was found at day 1 (Supplementary Fig. 1A–B). Similarly, glycemia was affected by the I/R and a significant drop in blood glucose was measured at day 1 after surgery for the vehicle group but not for GC7-treated mice (Supplementary Fig. 1C).

Renal fibrosis is a major late feature of I/R injury, reflecting the deleterious effects of earlier events. Herein, renal I/R led to the development of fibrosis at day 30, characterized by areas of type I and III collagen fibers deposits, highlighted by Sirius red staining (Fig. 1F, Supplementary Fig. 1E), and increased vimentin expression (marker of epithelial-mesenchymal transition), detected by immunostaining (Fig. 1G, Supplementary Fig. 1F), with a 3-4-fold increase compared to sham mice (Fig. 1F and G). As expected, analysis of GC7-treated kidneys drastically prevented fibrosis, trending towards normal values (Fig. 1F and G). This protection was correlated with a decreased expression of type-I collagen mRNA in GC7- compared to vehicle-treated mice (Supplementary Fig. 1D).

Altogether these results suggest that GC7 preconditioning led to a decrease of oxidative stress along with an increase of antioxidant enzyme activities during the early phase of kidney I/R, coupled to a faster functional recovery and limited late onset injury.

### 3.2 Rapid and sustained proteome reshaping upon GC7 treatment

**Fig. 2:**
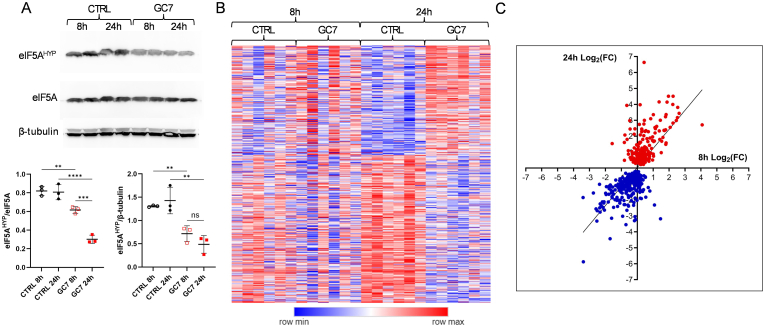
**Proteomic analysis of PCT cells treated with GC7.** In normoxia, PCT cells were treated with 30 μM of GC7 8 h and analyzed at the end of treatment or the day after (24h). (A) hypusination of eIF5A evaluated by Western blot. Plots display individual values and mean ± SEM. ∗p < 0.05, ∗∗p < 0.01, ∗∗∗p < 0.001, ∗∗∗∗p < 0.0001, One-way ANOVA and Tukey post-hoc test for multiple comparison. (B) Heat map and (C) correlative analysis of proteins modulated by the treatment at the different time points compared to untreated cells.

*In vivo* results suggested that GC7 pretreatment efficiently protects from I/R kidney injury, and potentially proximal tubule damage, the most sensitive to I/R injury and whose lesions contribute mainly to kidney failure. To validate this hypothesis, we have analyzed the effect of GC7 pretreatment on murine proximal convoluted tubule cells (PCT) with multiple approaches. First, we performed a deep comprehensive proteomic analysis of PCT either after 8 h of 30 μM GC7 or 24 h later, after a 16-h washout. As expected, the treatment inhibited eIF5A hypusination as well as the total cellular amount of hypusinated-eIF5A at 8h and inhibition persisted and was even amplified at 24h (Fig. 2A). Proteomic analysis revealed a strong modulation of proteins upon hypusination inhibition, with a large group (477) of significantly deregulated proteins (DEPs) induced after 24h. Importantly, these proteins were already deregulated in early timepoint (8h), with smaller amplitude, as shown by heatmap and correlation graph (Fig. 2B and C), demonstrating a rapid and incremental reprogramming of the proteome induced by GC7. As 8 and 24 h showed similar patterns, we decided to focus on the 24 h timepoint to evaluate stronger GC7 effects on the impact of anoxia/reoxygenation (A/R) stress.

**Fig. 3:**
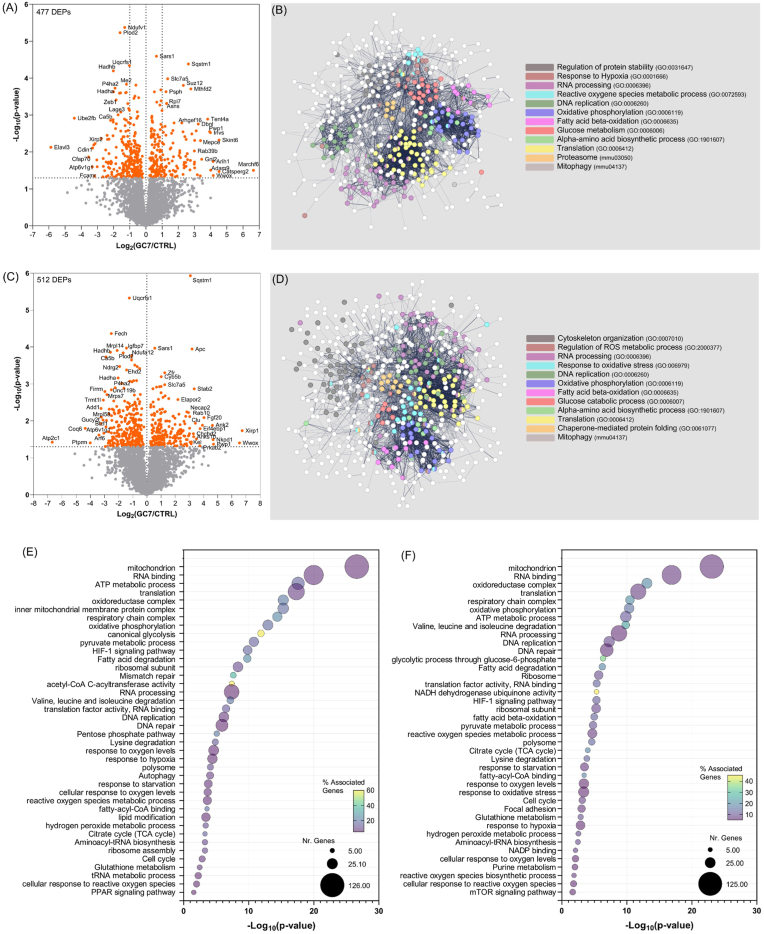
**Proteomic analysis of PCT cells after 8h treatment with GC7 and additional 16h washout (24h).** Changes in protein expression represented in volcano plots of GC7-treated cells under normal (A) or anoxia/reoxygenation (C) conditions. Network analysis of identified DEPs in normal (B) or anoxia/reoxygenation (D) conditions, highlighting interactions and clusters of proteins belonging to specific functional pathways. Pathway enrichment analysis of identified DEPs in normal (E) and anoxia/reoxygenation (F) conditions (showing 40 main pathways).

Thus, we designed this cellular A/R model to reproduce *in vivo* I/R stress: PCT cells were treated with GC7 for 8 h, washed out for 16 h, to mimic a preconditioning period, and then either subjected to 4 h of warm anoxia followed by 2 h of reoxygenation to mimic I/R or maintained under normoxia. Proteomic analysis of GC7-treated cells under normoxia and A/R revealed deep modifications in protein expression, as witnessed by a large set of proteins deregulated with a wide range of expressions (Fig. 3A–C). Network analysis of the DEPs upon GC7 treatment showed that the encoding genes are strongly associated with each other (networks in Fig. 3B–D; PPI enrichment p-value lower than 1.0e-16) suggesting an orchestrated change in protein expression rather than a random response. Indeed, deregulated proteins are grouped in specific functional clusters, associated with gene ontology annotated pathways (Fig. 3B–D, Supplementary Fig. 2A and B). For example, GC7 treatment resulted in a profound remodeling of the cell energy metabolic pathways, such as glucose and amino-acid metabolism, fatty acid β-oxidation and oxidative phosphorylation, suggesting a priming of the cell energy resources. Along down-regulated proteins highlighted by proteomic analysis, we found the glucose transporter SLC2A1 (GLUT1) (Supplementary Fig. 2; Supplementary Data 1, 2 and 3) confirming our previous results *in vitro* in PCT cells and *in vivo* in kidney [37]. We also observed an impact on RNA processing and proteostasis-related processes, as expected with hypusination inhibition. Interestingly, we obtained the same type of results *in vivo*, as analysis of protein expression in mouse renal cortex after 8 h of GC7 treatment (Sham-GC7 H3 and Sham-NaCl H3) showed a significant increase of sequestosome 1 (p62/SQSTM1) and seryl-tRNA synthase (SARS1) after GC7 treatment (Supplementary Fig. 3A). These proteins, which were the most up-regulated in PCT cells, are of interest as p62/SQSTM1 is a master regulator of autophagy, proteostasis and metabolism, and SARS1 a tRNA synthase crucial for protein translation and previously characterized as a target for anti-ischemic strategy in PCT cells and neurons [38]. Importantly, under normoxia, GC7 treatment already modulated the pathways of the cell response to hypoxia and ROS metabolism (as shown in clusters in Fig. 3B and Supplementary Fig. 2A), attesting hypusination relevant role in these defense mechanisms. When GC7-treated cells underwent A/R, these effects were maintained and similar functions impacted, demonstrating a significant impact on ROS and oxidative stress response, compared to anoxic control cells (Fig. 3D and Supplementary Fig. 2B). Such proteomic modulations in our cellular A/R model were similarly observed *in vivo* during IR. Indeed, immunohistofluorescence analysis of kidney submitted to IR shown that SARS1 was upregulated under GC7 treatment before (T0) and after IR (H3), as well as HADHA (hydroxyacyl-CoA dehydrogenase) which showed a strong downregulation *in vivo* at both time points (Supplementary Fig. 3B). HADHA is one of the most deregulated proteins after GC7 treatment under normoxia and A/R in PCT cells (Fig. 3A and B) and is the α-subunit of the Mitochondrial trifunctional protein (MTP) catalyzing mitochondrial β-oxidation of long-chain fatty acids. The β-subunit HADHB was also downregulated by GC7 treatment independently of stress (Fig. 3A and B).

Gene enrichment pathway analysis confirmed changes in these major functions driven by GC7, identifying 366 significantly enriched pathways in normoxia and 370 in A/R (top 40 pathways displayed in Fig. 3E and F), most of which belong to the mentioned macro-processes in the cells: cell metabolism, RNA processing, oxidative response. Indeed, in both normoxia and A/R, the analysis highlighted a notable remodeling of mitochondrial content, with around 125 mitochondrial proteins altered by GC7, correlated to changes in TCA cycle, fatty acid and glucose metabolism. The analysis also highlighted differential expression in proteins mediating DNA replication and repair, an activation of defense response and a stop in replicative potential.

In line with our previous observations, upon GC7 treatment we also identified upon GC7 treatment changes in processes related to the antioxidant response: impacting glutathione metabolism, hydrogen peroxide metabolism, response to ROS and to hypoxia (Fig. 3E and F).

Overall, our proteomic analyses showed that a short treatment with GC7 is able to induce deep and sustained proteome remodeling in the cell. Moreover, these changes in cell pathways were also maintained throughout A/R, suggesting that priming cells with GC7 before a stress gear them toward a beneficial and lasting expression profile, likely conferred through the observed modulation of the antioxidant defenses.

### 3.3 GC7 protects from oxidative stress during anoxia and reoxygenation and associated damage

To decipher GC7 impact on pathway modulations highlighted by proteomics analysis, we analyzed their sensitivity and adaptation to anoxia. As previously performed, PCT were treated with GC7 for 8 h followed by a washout period of 16 h. They were then submitted to anoxia for 4 h and reoxygenated for 2 h (A/R). As expected, pretreatment with GC7 led to approximately 50 % inhibition of eIF5A hypusination under normoxic conditions. (Supplementary Fig. 4A). Interestingly, 4-h A/R also strongly inhibited hypusinated eIF5A level, independently of GC7 pretreatment (Supplementary Fig. 4A).

**Fig. 4:**
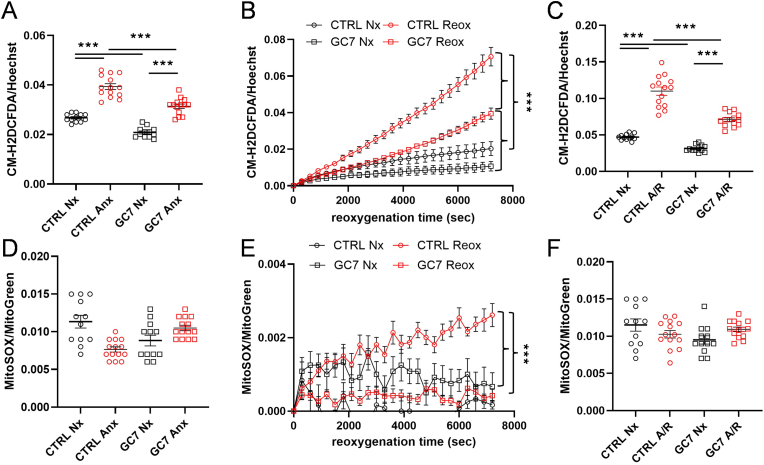
**GC7 decreases ROS production during anoxia and reoxygenation.** PCT cells were pretreated with 30 μM GC7 for 8 h and submitted 16 h later to 4 h of anoxia (A) and 2 h of reoxygenation (B, C). Fluorescence analysis assessing oxidative stress (CM-H2DCFDA probe, A-C) and mitochondrial ROS production (MitoSOX probe, D-F) were evaluated within live PCT cells at the end of anoxia (A, D), along reoxygenation (B, E) or cumulatively at the end of anoxia/reoxygenation (C, F). Hoechst 33342 (quantity of cells) and MitoGreen (quantity of mitochondria) were used to normalize CM-H2DCFDA and MitoSOX fluorescence signals respectively. Plots display individual values and mean ± SEM. Curves display the mean ± SEM, n = 12. ∗p < 0.05, ∗∗p < 0.01, ∗∗∗p < 0.001, ∗∗∗∗p < 0.0001, One- or Two-way ANOVA and Tukey post-hoc test for multiple comparison.

As expected, PCT submitted to 4 h of anoxia showed an increased CM-H2DCFDA fluorescence (Fig. 4A) revealing an increase in ROS levels, significantly dampened by GC7 treatment and this effect was preserved during and after reoxygenation (Fig. 4B and C). Hypusination inhibition prevented approximately 50 % of the oxidative stress produced by anoxia and/or reoxygenation (Fig. 4A and C). Interestingly, ROS production during anoxia or at the end of anoxia/reoxygenation did not correlate with mitochondrial ROS production as shown by MitoSOX signal, and this independently of GC7 pretreatment (Fig. 4D and F). Nevertheless, a slight increase in mitochondrial ROS production was found after reoxygenation only in untreated cells but did not seem to match the strong increase in oxidative stress found at the same time (Fig. 4E).

### 3.4 GC7 protects against oxidative stress and cell death induced by mitotoxic agents

**Fig. 5:**
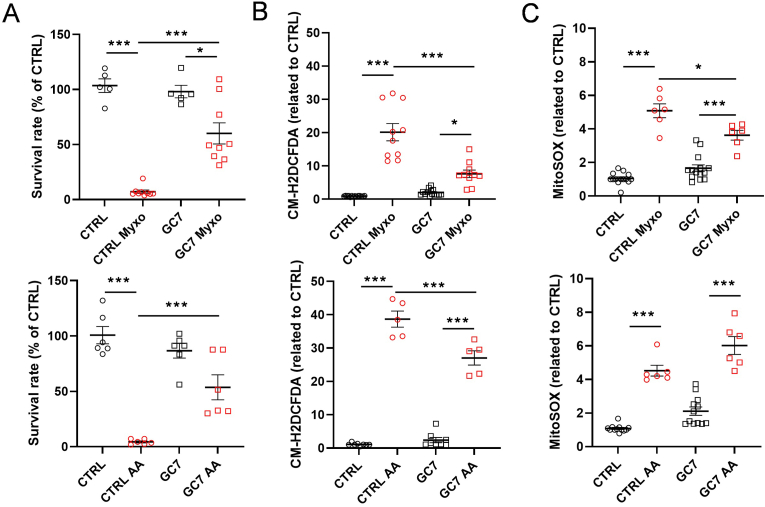
**GC7 protects from oxidative stress and cell death induced by mitotoxic treatment independently of mitochondrial modifications.** PCT cells were pretreated with 30 μM GC7 for 8 h and subjected 16 h later to Myxothiazol (Myxo, 1 μM) or Antimycin A (AA, 5 μM) treatment for 24 h. Cell survival (A) assessed by incorporation of calcein-AM (live cells) and ethidium homodimer (dead cells); dot plots displayed survival rate (Live/dead ratio in % of CTRL). Fluorescence analysis assessing oxidative stress (CM-H2DCFDA probe) (B) and mitochondrial ROS production (MitoSOX probe) (C), normalized by protein content. Plots display individual values and mean ± SEM. n = 5 to 12 as indicated. Dot plots display individual values and mean ± SEM. ∗p < 0.05, ∗∗p < 0.01, ∗∗∗p < 0.001, ∗∗∗∗p < 0.0001, One-way ANOVA and Tukey post-hoc test for multiple comparison.

To deepen our understanding of GC7 pretreatment on mitochondrial ROS production, we exposed PCT cells to various mitotoxic treatments known to disrupt respiratory chain complex III. Indeed, myxothiazol (Myxo) is a competitive inhibitor of ubiquinol at the Q_0_ site and antimycin A (AA) inhibits the oxidation of ubiquinol to ubiquinone at the Q_i_ site, both leading to mitochondrial ROS production. PCT treated with either compounds inevitably underwent cell death which was significantly prevented by GC7 treatment (Fig. 5A). For both compounds, GC7 protective effect was associated with a reduction in cellular oxidative stress (Fig. 5B) whereas mitochondrial ROS were significantly increased by the mitotoxic agents and poorly modified by GC7 treatment: GC7 pretreatment only slightly reduced mitochondrial ROS induced by Myxothiazol (Fig. 5C).

Overall, these results demonstrated that GC7 protected cells from deleterious effects of mitochondrial ROS without affecting their production, suggesting an involvement in antioxidant defenses.

### 3.5 GC7 driven antioxidant defense

ROS production is balanced by the cell's antioxidant defenses, and an increase in the latter could explain the protection against oxidative stress observed in GC7-pretreated PCT cells. We first analyzed Super-Oxide Dismutase (SOD) protein levels which synthesize H_2_O_2_ from superoxide anion (Supplemental Fig. 4B). While SOD1 (Cu^2+^/Zn^2+^-SOD, cytosolic) protein levels were equivalent in untreated and GC7 pretreated cells independently of A/R, SOD2 (Mn^2+^-SOD, mitochondrial) expression was downregulated by GC7 pretreatment in normoxic and short-anoxic conditions. Those results confirm the lower levels of SOD2 detected by the proteomic analysis in similar conditions (Supplementary Data 1).

**Fig. 6:**
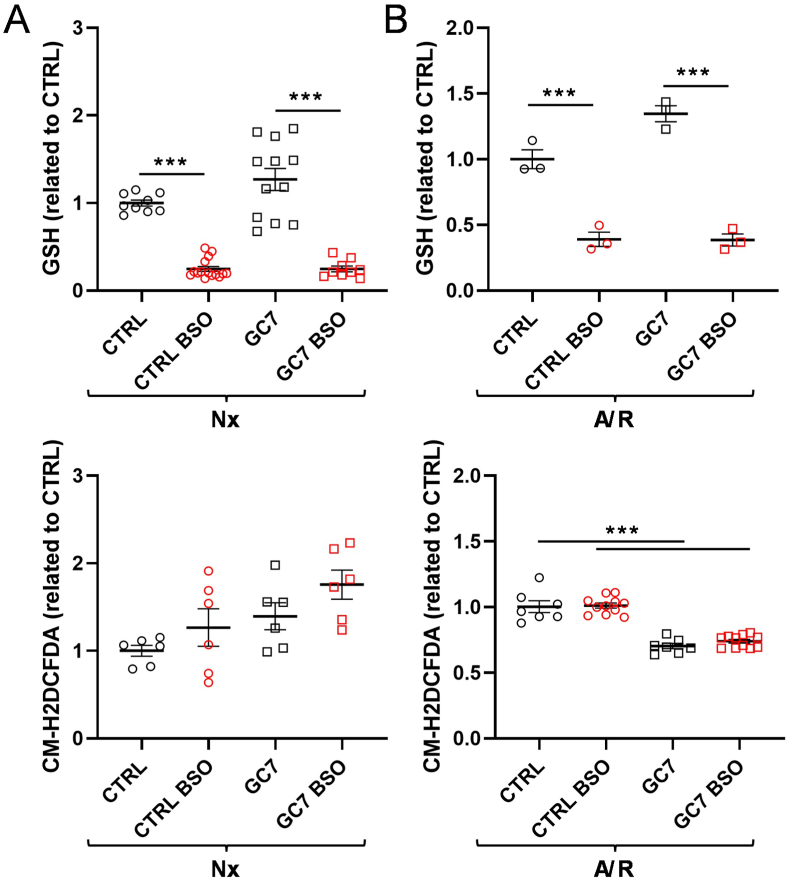
**Effect of GC7 pretreatment on GSH pathway.** PCT cells were pretreated with BSO 100 μM for 24 h, with or without GC7 30 μM during the 8 first hours (A–B). Cells were analyzed for GSH content and oxidative stress at the end of BSO treatment (A), after 4 h of anoxia followed by 2 h of reoxygenation (B). Fluorescence analysis assessing oxidative stress (CM-H2DCFDA probe) was evaluated within live PCT cells at the end of anoxia/reoxygenation (A/R). Total protein content was used to normalize GSH levels, while Hoechst 33342 was used to normalize the fluorescence signal on the quantity of cells. Plots display individual values and mean ± SEM. Curves display the mean ± SEM, n = 12. ∗p < 0.05, ∗∗p < 0.01, ∗∗∗p < 0.001, ∗∗∗∗p < 0.0001, One-way ANOVA and Tukey post-hoc test for multiple comparison.

We then investigated the effects of GC7 on glutathione (GSH/GSSG, reduced/oxidized) and catalase pathways, major players in H_2_O_2_ clearance. GC7 pretreatment did not alter the GSH content in PCT (Fig. 6A) and, interestingly, while 24 h exposure to 0.1 mM buthionine sulfoximine (BSO, an inhibitor of GSH synthesis) strongly reduced GSH levels in untreated and GC7 pretreated PCT, there was no effect on oxidative stress (Fig. 6A). This finding was also confirmed in cells subjected to 4 h of anoxia and 2 h of reoxygenation (Fig. 6B). GC7 pretreatment decreased CM-H2DCFDA fluorescence but BSO did not affect it, suggesting that *de novo* synthesis of GSH was not involved in GC7 protective effect.

**Fig. 7:**
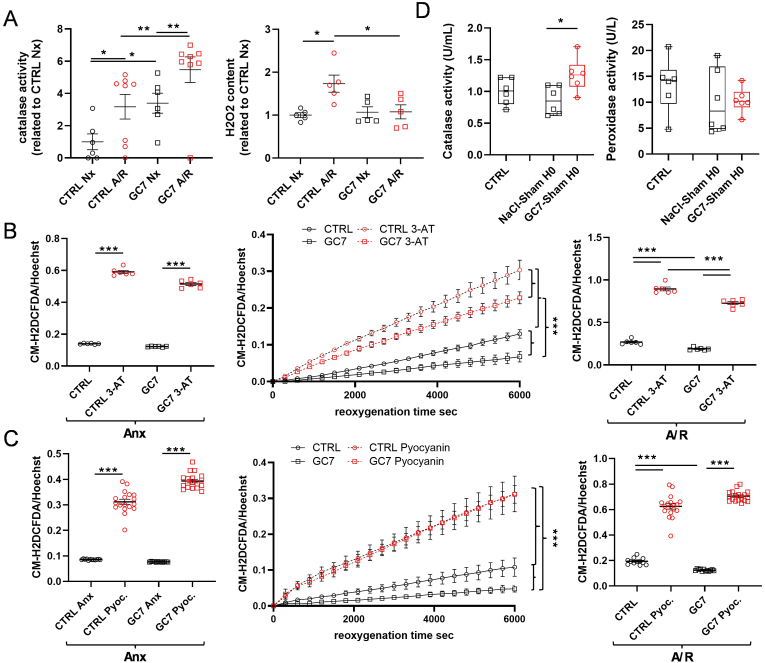
**Role of catalase in GC7 protective effect against oxidative stress.** (A–C) PCT cells were pretreated with 30 μM GC7 for 8 h and submitted 16 h later to 4 h of anoxia and 2 h of reoxygenation. (A) At the end of the stresses, catalase activity and H_2_O_2_ content were assessed and normalized to the total protein content. 1 h before anoxia, cells were treated with the catalase inhibitors 3-Amino-1,2,4-triazole (3-AT, 30 mM) (B) or pyocyanin (20 μM) (C), and oxidative stress (CM-H2DCFDA probe) was evaluated within live PCT cells at the end of anoxia (left panels), during reoxygenation (middle panels) or at the end of anoxia/reoxygenation (right panels). Hoechst 33342 was used to normalize fluorescence signal on the quantity of cells. (D) Mice were injected intraperitoneally with GC7 (3 mg/kg) or vehicle (NaCl) and subjected 4h later to sham procedure of IR (no nephrectomy and no renal ischemia) for 32 min. Catalase and peroxidase activities were evaluated in whole kidney lysates. Plots display individual values and mean ± SEM. Curves display the mean ± SEM, n = 12. Boxes displayed individual values and their distribution, mean, minimum and maximum. ∗p < 0.05, ∗∗p < 0.01, ∗∗∗p < 0.001, ∗∗∗∗p < 0.0001, One- or Two-way ANOVA and Tukey post-hoc test for multiple comparison (A–C) or Kruskal Wallis & Dunn's multiple comparison test (D).

Finally, we evaluated the last step in cell antioxidant mechanism, the catalase activity, to transform reactive H_2_O_2_ into H_2_O. Upon exposure to A/R (4 h anoxia/2 h reoxygenation), PCT exhibited a dramatic increase in catalase activity, exacerbated by GC7 pretreatment (Fig. 7A), a result corroborating the increased catalase activity found *in vivo* after IR and GC7 treatment (Fig. 1B). This is correlated with the decreased H_2_O_2_ levels in GC7 treated cells after A/R (Fig. 7A), which is not observed in control conditions. Interestingly, under resting conditions, PCT pretreated with GC7 exhibited a two-fold higher catalase activity (Fig. 7A). Such an increase in catalase activity was also observed *in vivo* in the kidneys of Sham GC7-treated mice when compared to Sham NaCl-treated animals at H0, a time point equivalent to the end of anoxia in IR mice (Fig. 7D).

Thus, we decided to challenge catalase involvement in GC7 protective effects using specific inhibitors. Indeed, inhibition of catalase activity by 30 mM of 3-Amino-1,2,4-triazole (3-AT) or 20 μM of pyocyanin led to an increase in CM-H2DCFDA fluorescence after anoxia and subsequent reoxygenation (Fig. 7B and C). These treatments led to a strong and even complete reversal of GC7 protective effect towards oxidative stress (3-AT and pyocyanin respectively, Fig. 7B and C). Importantly, the increased catalase activity observed following GC7 treatment and/or A/R was not due to an increased catalase protein level (Supplementary Fig. 4C). Moreover, this higher activity appeared to be linked to inhibition of eIF5A hypusination and not to a direct effect of GC7, as we found no modulation of the enzyme's reductase activity in the presence of GC7 (Supplementary Fig. 5A) or even antioxidant activity of GC7 itself (Supplementary Fig. 5B).These results demonstrated that catalase is a key component of GC7 protective effect against oxidative stress due to anoxia and reoxygenation.

### 3.6 GC7 protects cells against exogenous H_2_O_2_ stress

**Fig. 8:**
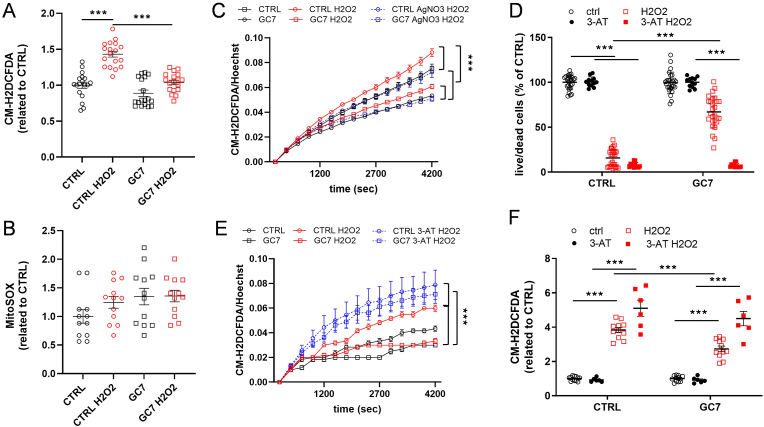
**Effects of GC7 pretreatment against H_2_O_2_-induced stress.** PCT cells were pretreated with 30 μM GC7 for 8 h and submitted 16 h later to various treatments. (A–B) Cells were submitted to H_2_O_2_ 10 mM for 20 min. (C–F) Cells were treated with (C) AgNO3 (5 μM, 1 h) to block aquaporin or with (D–F) catalase inhibitor 3-AT (30 mM, 30 min) and then by H_2_O_2_ (10 mM, 20 min). Cells were washed and analyzed immediately (A, B, C, E) or 6 h later (D, F). (A, C, E, F) Fluorescence analysis assessing oxidative stress (CM-H2DCFDA probe) and (B) mitochondrial ROS production (MitoSOX probe). Hoechst 33342 (quantity of cells) and MitoGreen (quantity of mitochondria) were used to normalize CM-H2DCFDA and MitoSOX fluorescence signals respectively. Cell survival analysis assessed by incorporation of calcein-AM (live cells) and ethidium homodimer (dead cells). Dot plots displayed survival rate (Live/dead ratio in % of CTRL). Curves display the mean ± SEM, n = 12. Dot plots display individual values and mean ± SEM. ∗p < 0.05, ∗∗p < 0.01, ∗∗∗p < 0.001, ∗∗∗∗p < 0.0001, One- or Two-way ANOVA and Tukey post-hoc test for multiple comparison.

To further characterize GC7 effects on catalase, independently from anoxia, we exposed PCT to 10 mM H_2_O_2_ for 20 min. This treatment induced, as expected, oxidative stress (Fig. 8A), independently from mitochondrial ROS production (Fig. 8B), and was prevented by GC7 preconditioning (Fig. 8A). To verify that oxidative stress was due to H_2_O_2_ entry into the cells, we pretreated cells 1h with 5 μM AgNO_3_, a known inhibitor of aquaporins (the main cellular importers of H_2_O_2_) [39]. Pretreatment of PCT with AgNO_3_ blunted oxidative stress following H_2_O_2_ treatment, and so, independently from GC7 (Fig. 8C). Finally, we showed that 6 h exposure to 30 mM H_2_O_2_ led to a massive cell death that could be prevented by GC7 pretreatment (Fig. 8D). Cell death was expectedly associated with a strong oxidative stress (Fig. 8F), prevented by GC7 and demonstrating again the protective effect of GC7 against oxidative stress induced cell death. Consistently, inhibition of catalase activity by 3-AT pretreatment (30 mM for 30 min before H_2_O_2_) reversed GC7 protective effects by preventing oxidative stress buffering following H_2_O_2_ exposure or 6 h later (Fig. 8E and F respectively) and by completely blunting pro-survival effect of GC7 after 6h of H_2_O_2_ treatment (Fig. 8D). Globally, our data demonstrated GC7 pretreatment efficient protection from H_2_O_2_ induced oxidative stress and cell death in PCT, via an upregulation of catalase activity.

## 4 Discussion

**Fig. 9:**
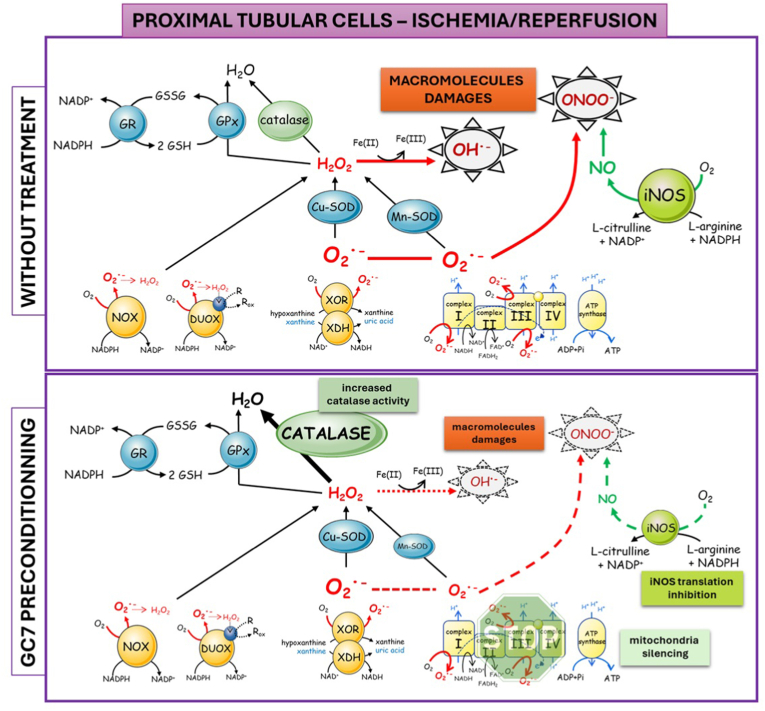
**Synthesis of the various productions and effects of ROS into proximal tubule cells during Ischemia/reperfusion and the evidence found for the same actors after GC7 treatment.** GR, glutathione reductase; GPx, glutathione peroxidase; NOX, NADPH oxidase; DUOX, dual oxidase; XOR, xanthine oxidoreductase; XDH, xanthine dehydrogenase; SOD, superoxide dismutase; iNOS, inducible nitric oxide synthase.

I/R, an unavoidable and inherent step of transplantation procedure, causes excessive cellular ROS. The resulting oxidative stress, when facing an insufficient antioxidant system, can overcome it and result in lesions, responsible of the main graft injury, particularly in the kidney [30] (Fig. 9). Through this study, we evidenced that the inhibition of the unusual eIF5A hypusination, via GC7, a very potent specific inhibitor of this pathway with pleiotropic effects, was a very efficient way to prevent short- and long-term damages associated with I/R and A/R, respectively in *in vivo* and *in vitro* models. Particularly, this protective effect is conferred by an improved cellular antioxidant system and therefore cells’ capacity to defend themselves from I/R and A/R associated oxidative stress and subsequent macromolecular damage (Fig. 9). This allows cells to survive to ischemic stress, as previously suggested in the porcine models of autotransplantation and allotransplantation [25,26].

Ischemia preconditioning (IPC) consists in applying brief and repeated occlusions on an organ artery to simulate sequential I/R, promoting organ's reprogramming and granting protection from future I/R associated lesions [40]. Specifically, IPC has been shown to activate signalling pathways and associated enzyme activities leading to modulation of redox function and energetic metabolism [41]. In this context, we decided to investigate GC7 protective role against oxidative stress and surprisingly, we evidenced that short-anoxia led to a strong inhibition of eIF5A hypusination. Hypusination second enzymatic step, hydroxylation of deoxyhypusine by DOHH, requires oxygen, thus it is not surprising to detect a decrease in hypusination during anoxia as previously demonstrated in yeast [28]. In our work, eIF5A hypusination inhibition at the time of the stress seemed unrelated to any protective effect, differing from a preconditioning hypusination inhibition due to GC7 pretreatment which is also a valuable preconditioning strategy to protect cells against I/R. In this regard, IPC and GC7 preconditioning both lead to highly valuable and similar protective mechanisms, including sustained catalase activity [42]. We can therefore hypothesize that during IPC, ischemic phases lead to sequential and/or additive inhibition of eIF5A, an effect reproduced by GC7. This hypothesis could finally explain the mechanism behind ischemic protection by IPC protocols used in clinic, related to the hormesis concept but it would require further studies to be proven.

Here we report, as expected, that increased ROS production in response to A/R was balanced by the cell antioxidant defense. In this setting, we clearly demonstrated that catalase was the main defense mechanism, correlated by its high expression in proximal tubular cells [43]. Interestingly, we did not evidence SOD-1 and -2 upregulation, or neosynthesis of GSH, which are other classical ROS detoxification systems. This last result could be explained by the fact that GSH is a key antioxidant in renal cell defense, especially for buffering oxidizing species generated by endoplasmic reticulum (ER) stress [9]. ER stress is another deleterious event observed during I/R, but it is also largely attenuated by GC7 treatment [44] and therefore does not require a downstream antioxidant system.

Finally, the absence of eNOS and iNOS in PCT proteomic profiling (Supplementary Data 1), confirmed by the fact that *Nos1* and *Nos2* are not or barely expressed in proximal tubule cells in physiological conditions [43], suggests that proximal tubule cells seem unable to produce NO. Thus, A/R induced PCT cell-death seems independent from RNS, neither from eNOS uncoupling nor from iNOS overexpression, which are the products of the NO reaction with ROS including the highly damaging peroxynitrite [45]. We can thus hypothesize that peroxynitrite produced during kidney I/R, *in vivo,* could originate from NO produced by other interstitial cells, such as immune or endothelial cells, that can react with ROS in these or adjacent epithelial cells.

Proteomic pathway analysis in response to hypusination inhibition by GC7 identified a large cohort of proteins affected, both rapidly upon treatment and with an effect lasting even after drug washout. Identified proteins are involved in the regulation of several processes in the cell. As one could expect from inhibition of eIF5A function, we observed deregulated RNA-processing and RNA-binding as well as proteins involved in polysome compartments. However, we did not observe a generalized loss of protein translation. Instead, we observed a concerted modulation of proteins belonging to specific pathways, interconnected in functional clusters, especially regarding metabolic processes. Importantly, mitochondrial content was significantly deregulated upon GC7 treatment in normoxic or anoxic conditions, with changes in OXPHOS and glucose metabolism, confirming the metabolic shift previously described [12,26,27] but insufficient to explain the broader GC7 effects. Among other pathways of interest, we analyzed the differential expression of proteins involved in autophagy since previous work implicated polyamines and eIF5a hypusination in the autophagic turnover and lymphocytes senescence [46]. Along these proteins, we found that p62/SQSTM1 was the most upregulated proteins after GC7 treatment *in vitro* and *in vivo*. SQSTM1 could be pivotal in GC7 effect as it is involved in proteostasis, metabolism and autophagy [47] three pathways modulated by eIF5A hypusination [29]. However, whether autophagy contributes to the protective effects of GC7 in PCT and more broadly to kidney transplantation still needs to be investigated. Of note, our proteomics analysis also highlighted several cell's networks involved in the oxidative stress defense, for example in response to oxygen levels, HIF-1α signalling pathway, hydrogen peroxide metabolism, etc … Importantly, GC7-pretreatment induced a lasting reprogramming of these pathways, even after exposure to A/R, revealing their critical importance and a signature of GC7 treatment effects, a property we further dissected in ad-hoc experiments. Characterization of the different origins of ROS production (mitochondrial *vs*. non-mitochondrial) along with anoxia and reoxygenation allowed us to improve our knowledge about GC7 protective effects and identify downstream effectors modulated by hypusination.

Originally, we hypothesized that mitochondrial silencing and metabolic shift induced by GC7 was the main cause of its protective effect against I/R injury and especially oxidative stress [26]. While oxidative to glycolytic shift may protect cells from both oxygen and nutrient deprivation by maintaining a sufficient ATP production [26,37], involvement of mitochondria and particularly, a decrease in activity, was not sufficient to confer protection against oxidative stress. Thus, this result really defines GC7 as a compound with distinct protective properties, different from already described molecules conferring kidney (or other organs) a protection from I/R by limiting or buffering mitochondrial ROS production, especially during reperfusion [12]. Specifically, our results demonstrated that the GC7 inhibition of mitochondria activity alone cannot explain the strong effect on oxidative stress, corroborated by a decreased SOD2 expression without accumulation of mitochondrial ROS following GC7 pretreatment.

A key finding of this work is the central role of catalase: increased catalase activity by GC7 treatment, independently of stress, conferred cell tolerance to oxidative stress while inhibition of catalase rapidly resulted in oxidative stress and cell death. Catalase activity is regulated at various levels including mRNA expression, protein stability and activity itself [48]. In our hands, we have not been able to correlate the increase of catalase activity with mRNA or protein overexpression upon GC7 pretreatment. Similarly, we found no direct effect of GC7 on catalase activity, nor any GC7 activity similar to that of catalase that might explain higher H_2_O_2_ clearance. Catalase is a complex enzyme and can be transformed in the inactive Compound II through interaction with H_2_O_2_, a deleterious reaction prevented by the binding of NADPH to catalase [49]. We have previously demonstrated that PCT treated with GC7 showed an increased glucose oxidation, mainly for ATP synthesis through anaerobic glycolysis, but we strongly suspect an increased consumption of glucose through the pentose phosphate pathway, which is the main source of NADPH in cells [37]. Therefore, we can hypothesize that under anoxia, while mitochondrial NADPH synthesis is disrupted, the pentose phosphate pathway dependent NADPH synthesis will contribute to maintain cellular antioxidant defences. Interestingly, GC7 treated PCT showed a sustained expression of SHMT2 (serine hydroxymethyltransferase 2 (SHMT2)) and especially of MTHFD2 (methylene-tetrahydrofolate dehydrogenase 2), two key proteins involved in NADH and NADPH mitochondrial synthesis from serine catabolism independently from mitochondrial respiratory chain [[50], [51], [52]]. Thus, PCT could use serine as an alternatively source of NADPH for their antioxidant defences.

Understanding where and when oxidative stress occurs during renal I/R, and how to manage it, is crucial knowledge in the field of organ transplantation. Indeed, ischemic-oxidative stress associated lesions will lead to a destabilization of the epithelium and tubular necrosis. Both will result in an episode of acute renal failure and a delayed functional recovery, as well as establishment of an inflammatory and fibrogenic/fibrotic environment, major determinants of graft failure, rejection and loss [1,6,8,11,12,53]. As transplantation requires an inevitable sequence of I/R, the donor's condition will therefore dramatically influence the extent of I/R lesions. Obviously, chances of a functional recovery of the organ will be higher if the donor is alive, young and healthy [53], particularly because planning surgery from a living donor allows for shorter ischemia times, and therefore a better preservation of organ quality [54]. Unfortunately, the current situation combining increased conditions requiring a transplantation and organ shortage calls for the use of donors with extended criteria (marginal donors). Those donors, in particular elderly ones, present condition generally highly favourable to a higher oxidative stress [55,56]. Thus, GC7 may be considered an effective candidate for protecting organs of at-risk donors to limit oxidative stress and therefore the inherent ischemic damage thus promising an increase in graft success.

## 5 Conclusions

Herein, we demonstrated that the anti-ischemic effects of GC7 in epithelial cells from the kidney proximal tubule are due to several pathways such as improved antioxidant defences, a metabolic shift and a downregulation of mitochondrial activity. These modulations occur early after treatment and preserve organ function after I/R, making GC7 a strong candidate for use in the clinic to limit ischemic damage in donors sensitive to oxidative stress, particularly circulatory-dead as well as marginal and extended criteria donors. GC7 usage could improve both the number of organs available to transplantation and their quality. In addition, this study clarifies the anti-ischemic potential of hypusination inhibition and highlights specific modulation of pathways involved in redox homeostasis of cells, integrating the metabolic role of GC7 with its antioxidant potential, certainly through the promotion of the pentose phosphate pathway. The origin of ROS after I/R remains unclear, although as large producers and highly sensitive to I/R lesions, tubular cells are not the only producer and therefore, cells in their vicinity, possibly immune cells, must participate to those nefarious effects.

## CRediT authorship contribution statement

**Marc Cougnon:** Conceptualization, Formal analysis, Investigation, Supervision, Validation, Visualization, Writing – review & editing. **Sébastien Giraud:** Conceptualization, Formal analysis, Investigation, Methodology, Supervision, Validation, Visualization, Writing – original draft. **Maria Materozzi:** Conceptualization, Data curation, Formal analysis, Methodology, Validation, Writing – review & editing. **Elisa Allart:** Formal analysis, Investigation, Visualization. **Isabelle Rubera:** Formal analysis, Investigation, Methodology, Validation, Writing – review & editing. **Claire Mackowiak:** Investigation, Visualization. **Gisèle Jarretou:** Formal analysis, Investigation, Methodology. **Nadège Boildieu:** Investigation, Visualization. **Virginie Ameteau:** Investigation, Visualization. **Estelle Lemarié:** Investigation, Visualization. **Hajar Ouahmi:** Investigation, Visualization. **Nicolas Melis:** Formal analysis, Investigation, Methodology, Validation, Writing – original draft. **Mallorie Poet:** Investigation, Methodology, Validation. **Christophe Duranton:** Investigation, Methodology, Validation, Visualization. **Luc Pellerin:** Methodology, Project administration, Supervision, Visualization, Writing – review & editing. **Laurent Counillon:** Conceptualization, Formal analysis, Supervision. **Marina Shkreli:** Investigation, Methodology, Supervision, Validation, Visualization, Writing – review & editing. **Michel Tauc:** Conceptualization, Formal analysis, Funding acquisition, Investigation, Methodology, Project administration, Supervision, Validation, Visualization, Writing – review & editing. **Thierry Hauet:** Formal analysis, Funding acquisition, Methodology, Project administration, Supervision, Validation, Writing – review & editing. **Didier F. Pisani:** Conceptualization, Data curation, Formal analysis, Funding acquisition, Investigation, Methodology, Project administration, Supervision, Validation, Visualization, Writing – original draft.

## Declaration of competing interest

The authors declare that they have no known competing financial interests or personal relationships that could have appeared to influence the work reported in this paper.

## Appendix A Supplementary data

Supplemental Figure 1 Related to Fig. 1. Blood parameters and collagen RNA expression in mice submitted to renal Ischemia/Reperfusion. Mice were injected intraperitoneally with GC7 (3 mg/kg) or vehicle only (NaCl) and subjected 4h later to left nephrectomy and right renal ischemia for 32 min followed by reperfusion. (A) Blood creatinine, (B) blood urea (BUN) and (C) glycaemia evaluated at indicated day with D0 = Ischemia/Reperfusion (IR). (D) mRNA relative expression (to normal kidneys) of collagen evaluated in kidneys sampled 30 days after I/R (IR D30). Boxes displayed individual values and their distribution, mean, minimum and maximum. ∗p < 0.05, ∗∗p < 0.01, ∗∗∗p < 0.001, ∗∗∗∗p < 0.0001, Kruskal Wallis & Dunn's multiple comparison test or Mann-Whitney's comparison test for 2 groups. Total RNAs from mice renal tissues (frozen samples of kidney cortex collected at day 30 after ischemia-reperfusion) were extracted with NucleoSpin RNA Plus kit (Macherey-Nagel, France) and reverse transcription was performed with qScript cDNA Supermix kit (QuantaBio, France) following the manufacturer's recommendations. Real-Time PCR assays were carried out on a RotorGene-Q with RotorGene Syber Green PCR kit (Qiagen, France) following the manufacturer's recommendations. DNA primers were designed using Primer-BLAST and OligoAnalyser (Integrated DNA Technologies Inc), primers sequences are RPS5 (F: GAGCACTGATGACGTGCAGA, R: ATGGGACATTGTGCTTTGCG) and Collagen type I (F: CCCATTGACCTCTTCCACCT, R: AGCTGAAGAATCCACCACCA). RNA expression was expressed as Relative Fold Change to control kidney, determined with the Pfaffl method using Qbase + software (Biogazelle). Ribosomal Protein S5 (RPS5) gene was chosen as the most stable reference gene determined with the geNorm algorithm. (E-F) Histology analysis of kidney sections at day 30 after I/R (IR D30). (E) Sirius red staining visualized by normal light to evaluate global collagen fibers (magnification ×40) and (F) Vimentin immunostaining visualization by normal light (magnification ×100). Supplemental Figure 1

Supplementary Figure 2 Related to Fig. 3. Detailed network analysis of GC7-treated cells under normal (A) or anoxia (B) conditions showing gene name and related functional pathways. Supplementary Figure 2

Supplemental Figure 3 Related to Fig. 3. Mice were treated with 30 μM GC7 for 4 h (T0) and then subjected to unilateral kidney ischemia and 3 h of reperfusion (IR-H3) or equivalent sham procedure (Sham-H3). (A) Proteins were extracted from dissected kidney cortex and used for Western blot analysis of SARS1 and p62/SQSTM1 protein levels. β-actin was used as loading control. Dot plots display individual values and mean ± SEM. n = 6. ∗p < 0.05, ∗∗p < 0.01, Mann-Whitney test. (B) Immunohistofluorescence analysis of SARS1 and HADH on cortical kidney section before or after IR. Proteins were detected in green and nuclei in blue (DAPI) (Scale bar 100 μm). Supplemental Figure 3

Supplemental Figure 4 Related to Fig. 4, Fig. 6, Fig. 7. PCT cells were pretreated with 30 μM GC7 for 8 h and subjected 16 h later to 4 h of anoxia and 2 h of reoxygenation. (A) Western blot analysis of hypusine and eIF5A levels, (B) of SOD1 and SOD2 and (C) of Catalase cell levels. β-tubulin was used as loading control. Dot plots display individual values and mean ± SEM. n = 3. Plots display individual values and mean ± SEM. ∗p < 0.05, ∗∗p < 0.01, ∗∗∗p < 0.001, ∗∗∗∗p < 0.0001, One-way ANOVA and Tukey post-hoc test for multiple comparison. Supplemental Figure 4

Supplemental Figure 5 Related to Fig. 7. H_2_O_2_ reduction was assessed in (A) the presence or (B) the absence of various concentrations of catalase and with or without 30 μM of GC7. Dot plots display individual values and mean ± SEM. n = 4. ∗∗∗p < 0.001, ∗∗∗∗p < 0.0001, (A) One-way ANOVA and Tukey post-hoc test for multiple comparison or (B) Mann-Whitney test. Supplemental Figure 5
