# Supplementary material for: Inhibition of eIF5A hypusination enhances antioxidant defense to prevent kidney Ischemia/Reperfusion injury
Source: Redox Biol. 2025 Aug 6;86:103814. doi: 10.1016/j.redox.2025.103814 (PMC12357257; doi:10.1016/j.redox.2025.103814)

Supplementary Figure 1.

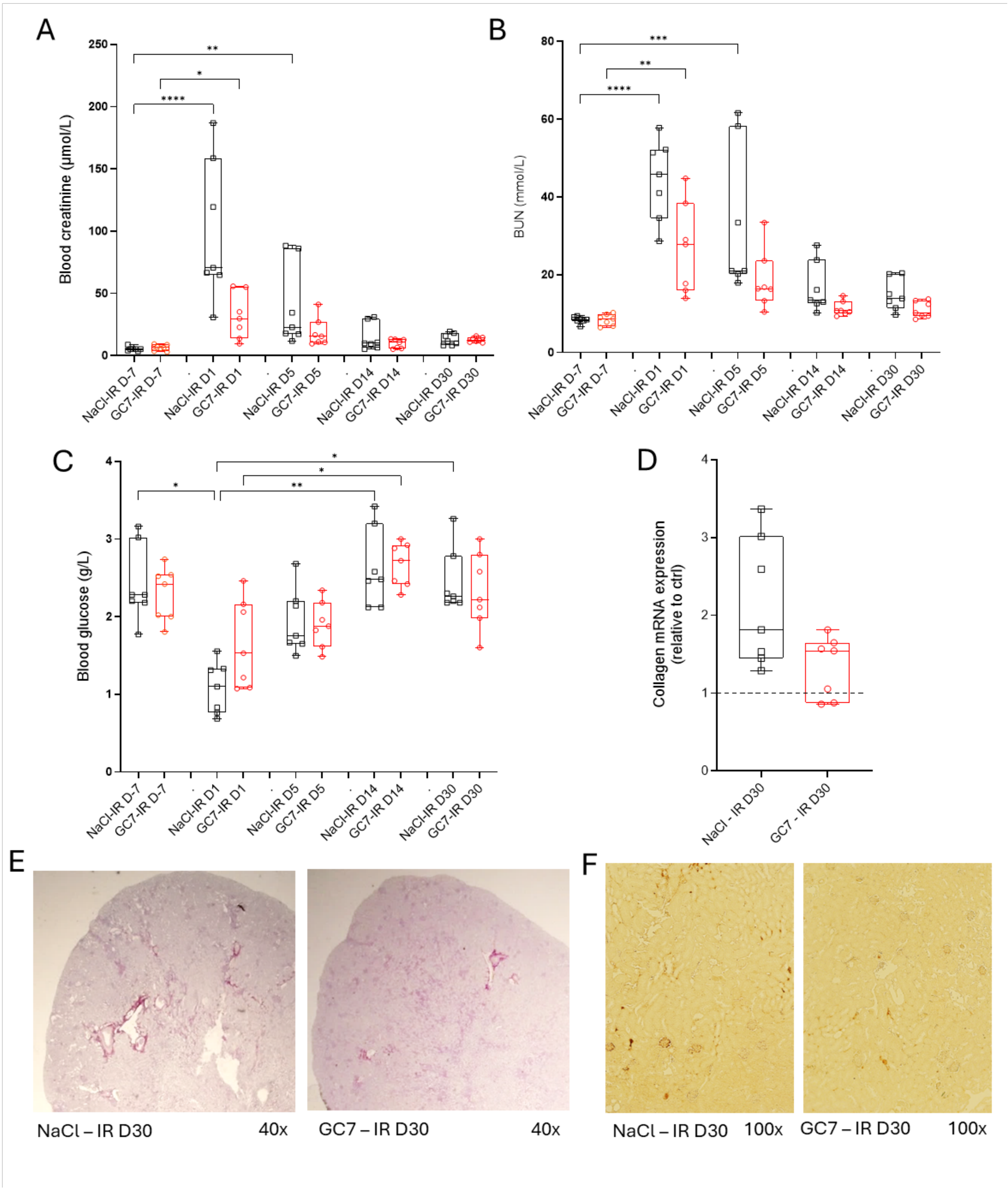

Supplementary Figure 2.

(A)

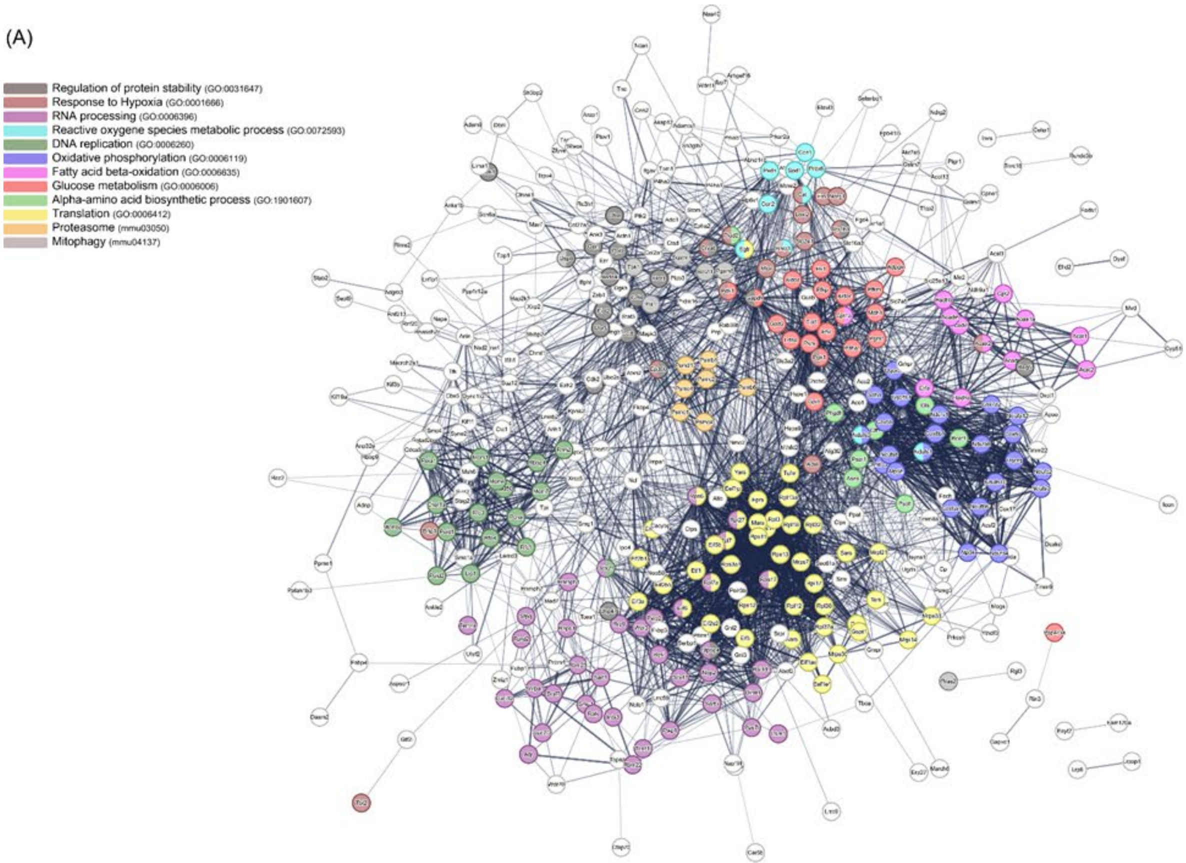

(B)

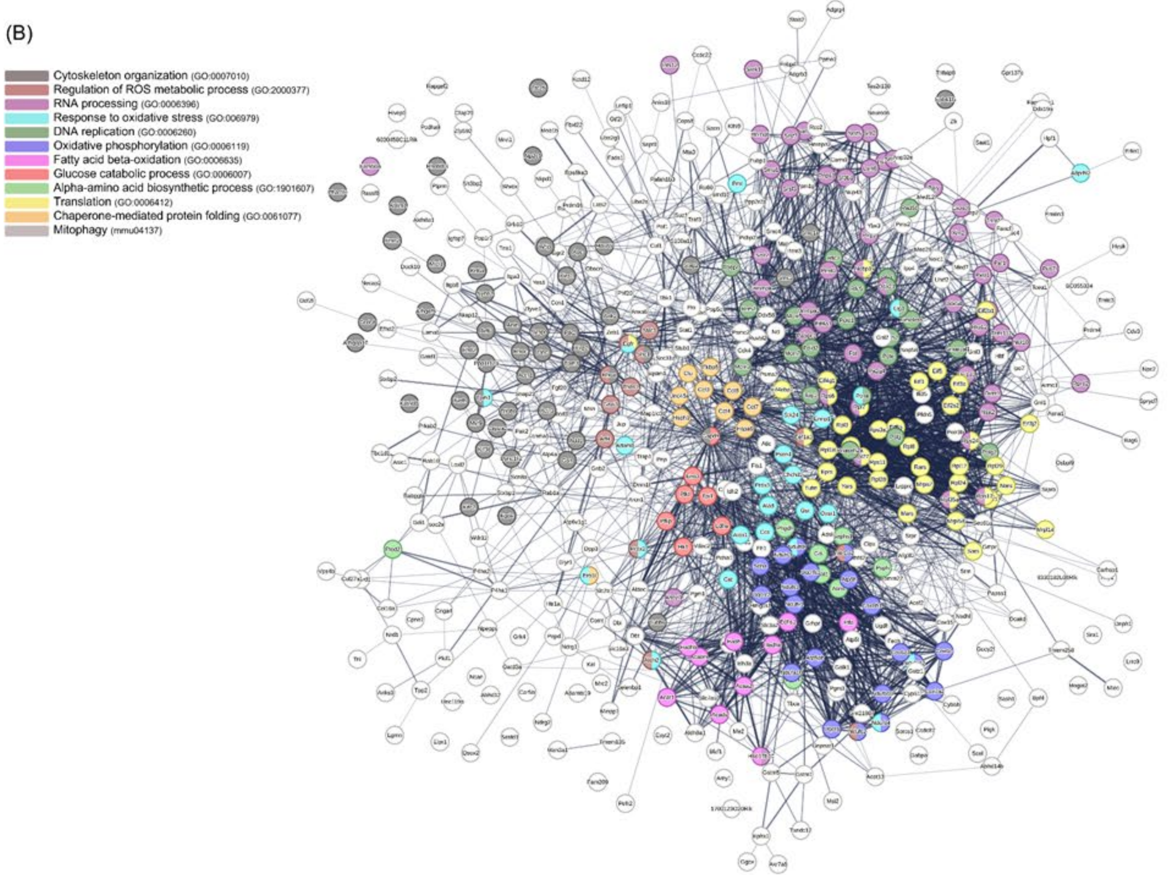

Supplementary Figure 3.

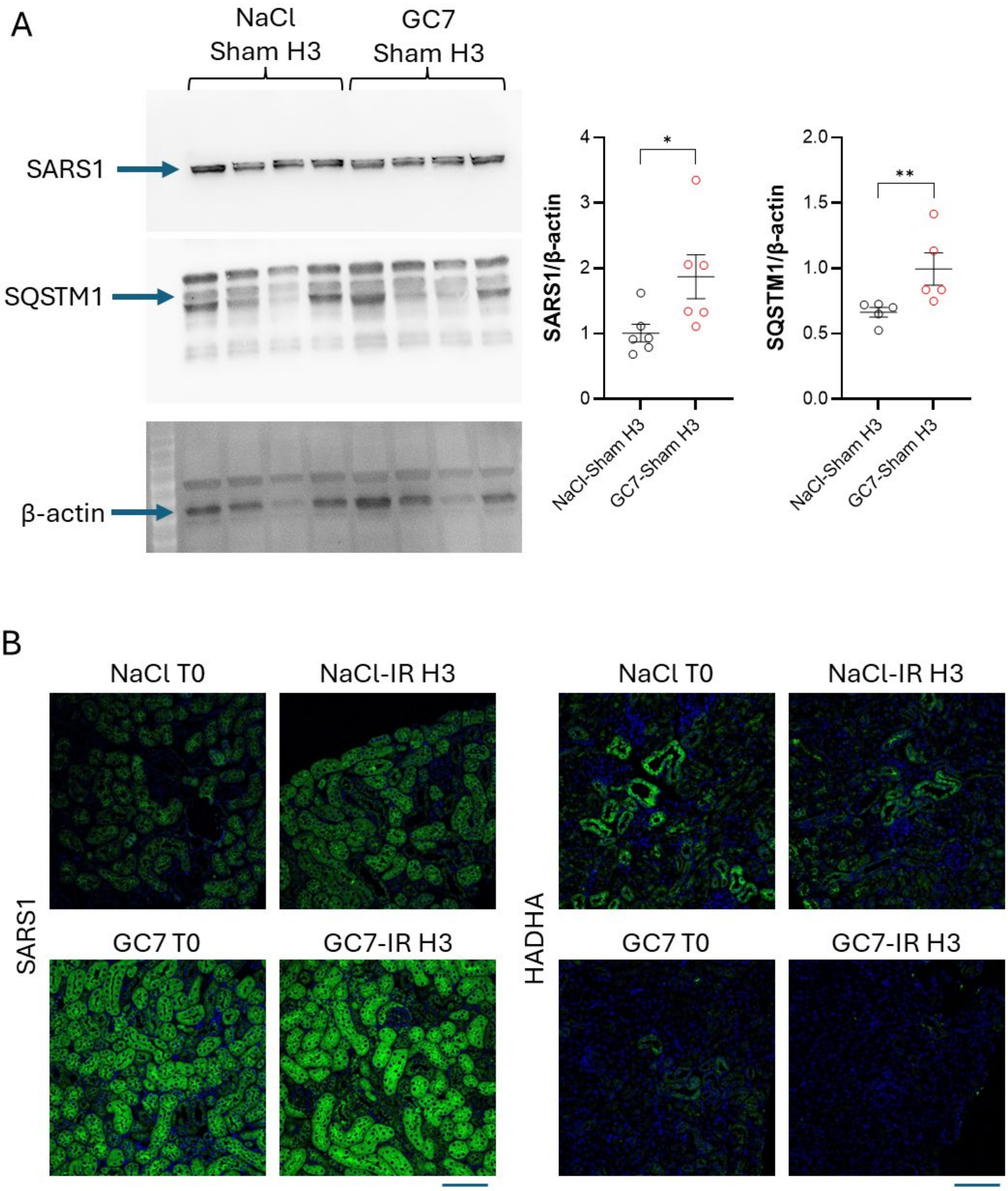

Supplementary Figure 4.

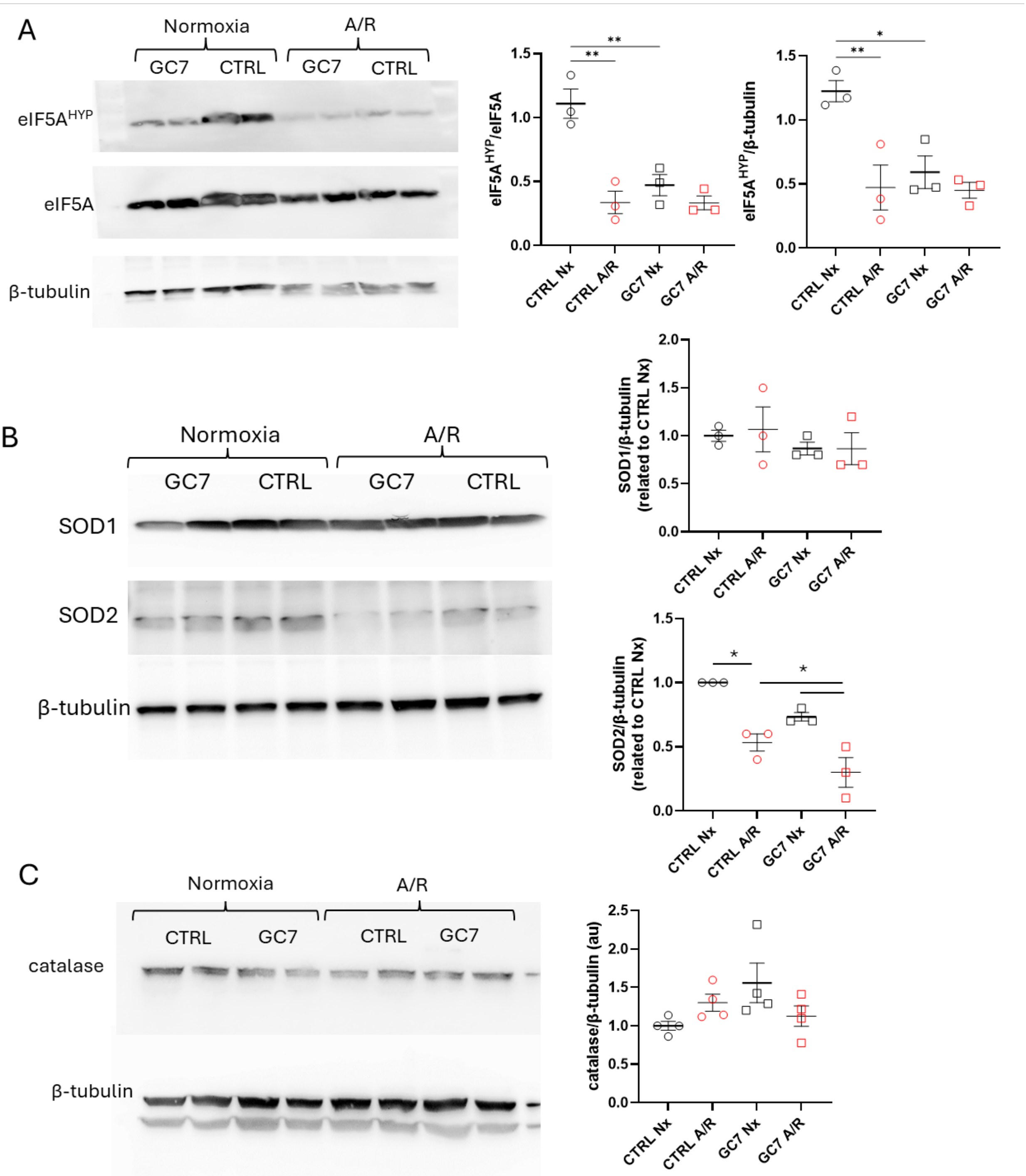

Supplementary Figure 5.

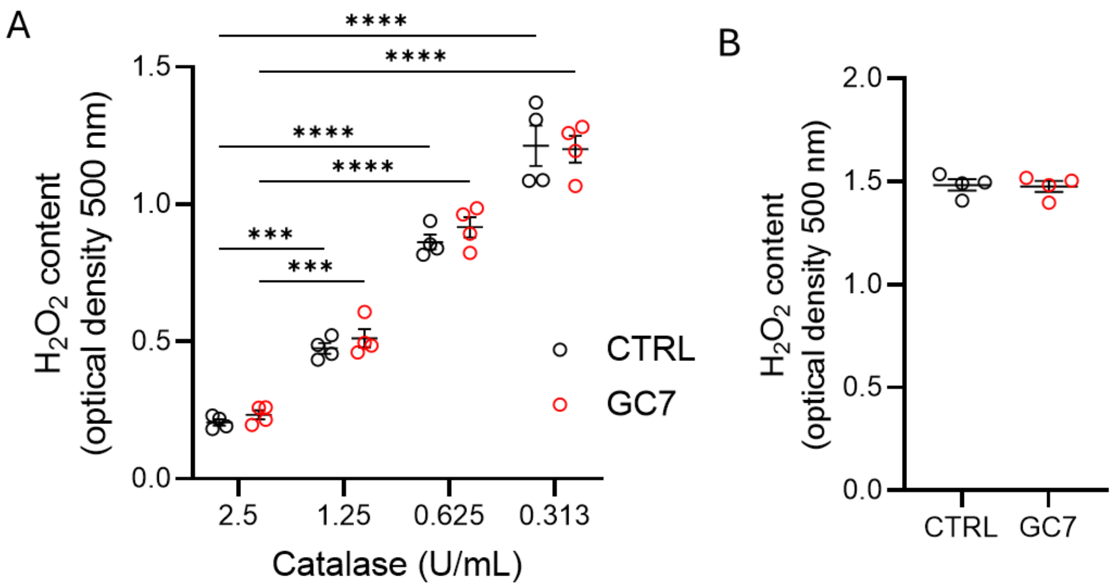

Supplement: Supplemental Figure 4 — Related to Fig. 4, Fig. 6, Fig. 7. PCT cells were pretreated with 30 μM GC7 for 8 h and subjected 16 h later to 4 h of anoxia and 2 h of reoxygenation. (A) Western blot analysis of hypusine and eIF5A levels, (B) of SOD1 and SOD2 and (C) of Catalase cell levels. β-tubulin was used as loading control. Dot plots display individual values and mean ± SEM. n = 3. Plots display individual values and mean ± SEM. ∗p < 0.05, ∗∗p < 0.01, ∗∗∗p < 0.001, ∗∗∗∗p < 0.0001, One-way ANOVA and Tukey post-hoc test for multiple comparison. [file mmc4.pdf]
