# Supplementary material for: Inhibition of eIF5A hypusination enhances antioxidant defense to prevent kidney Ischemia/Reperfusion injury
Source: Redox Biol. 2025 Aug 6;86:103814. doi: 10.1016/j.redox.2025.103814 (PMC12357257; doi:10.1016/j.redox.2025.103814)

Whole blots for Figure 2A

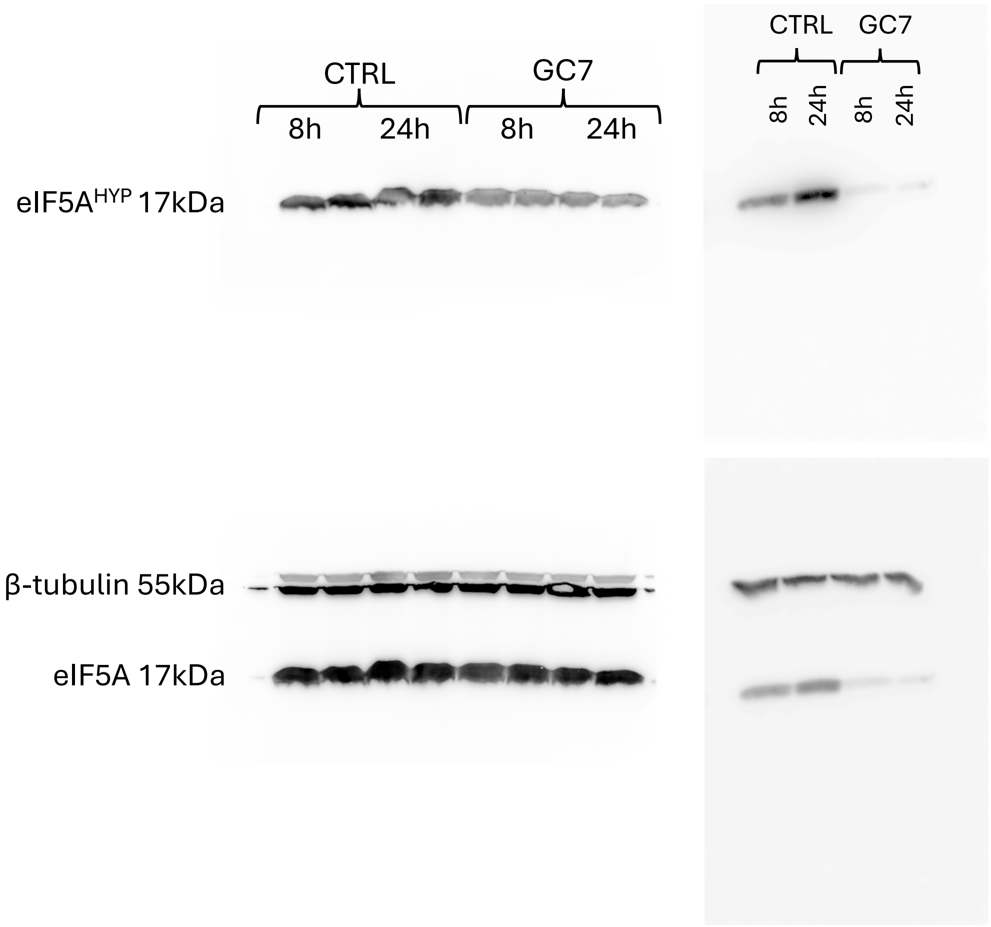

Whole blots for Supplementary Figure 3

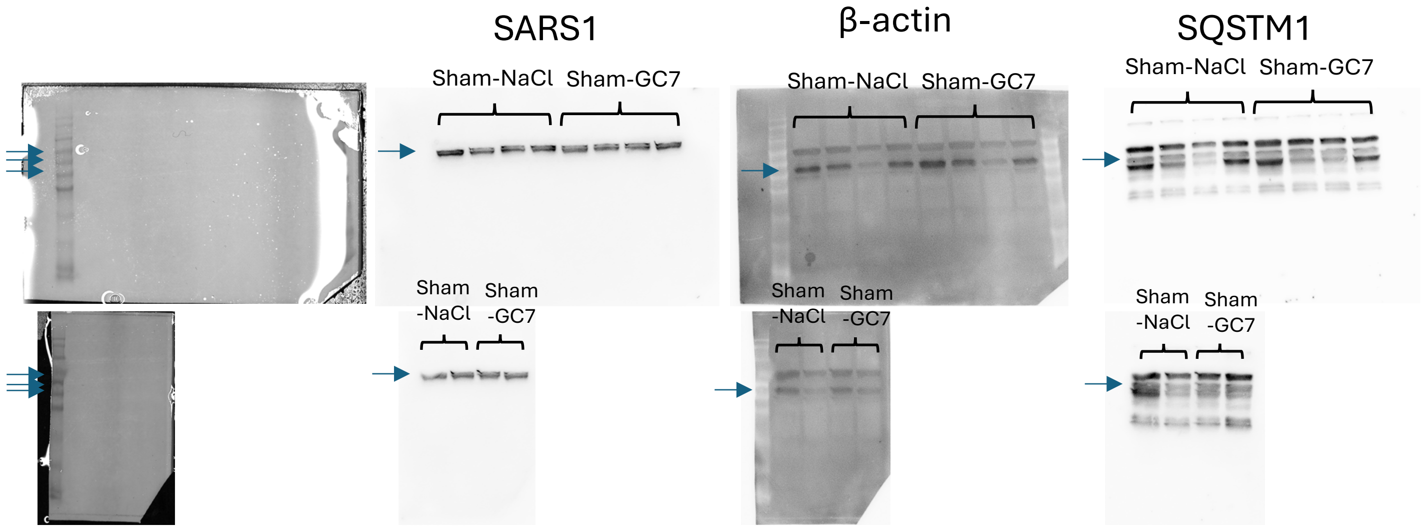

Whole blots for Supplementary Figure 4A

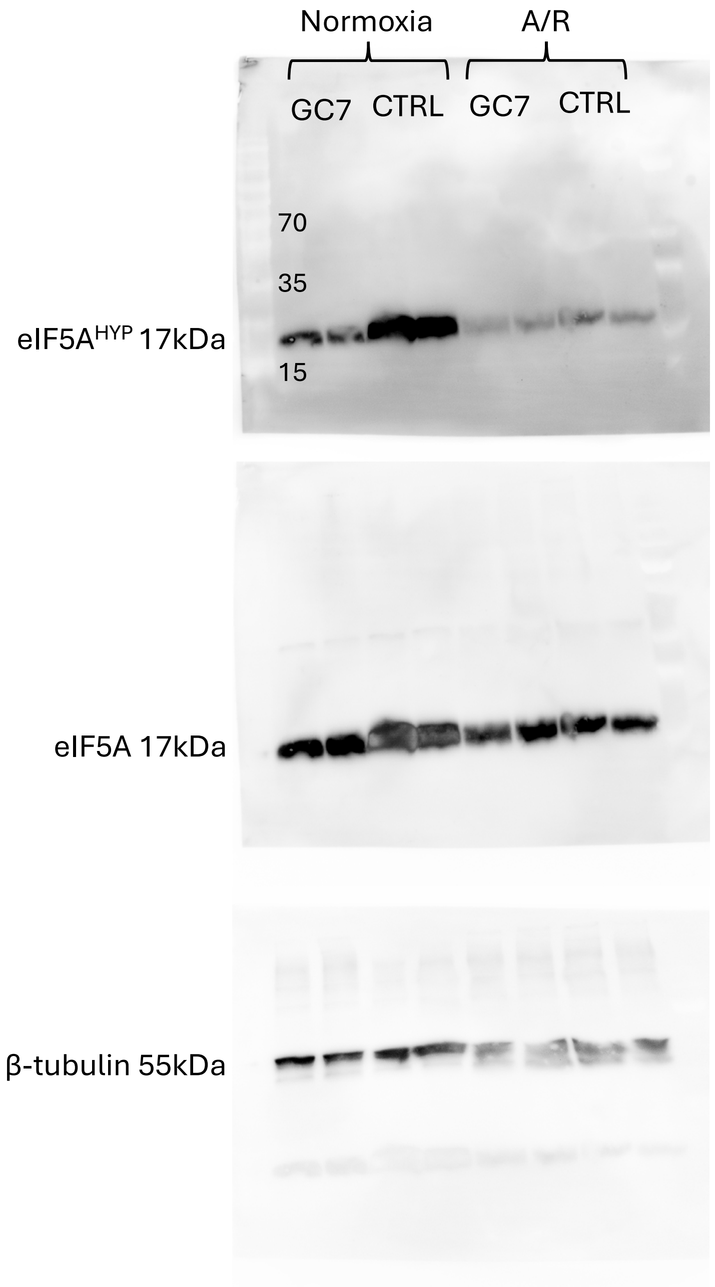

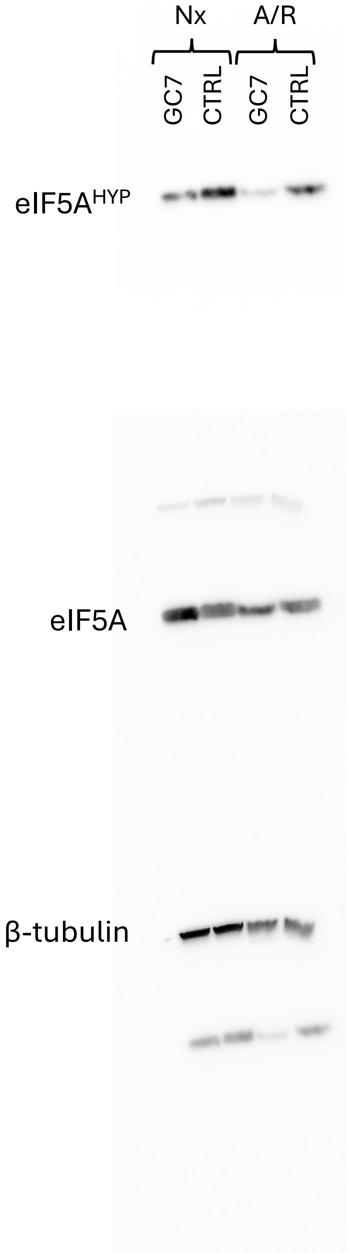

Whole blots for Supplementary Figure 4B

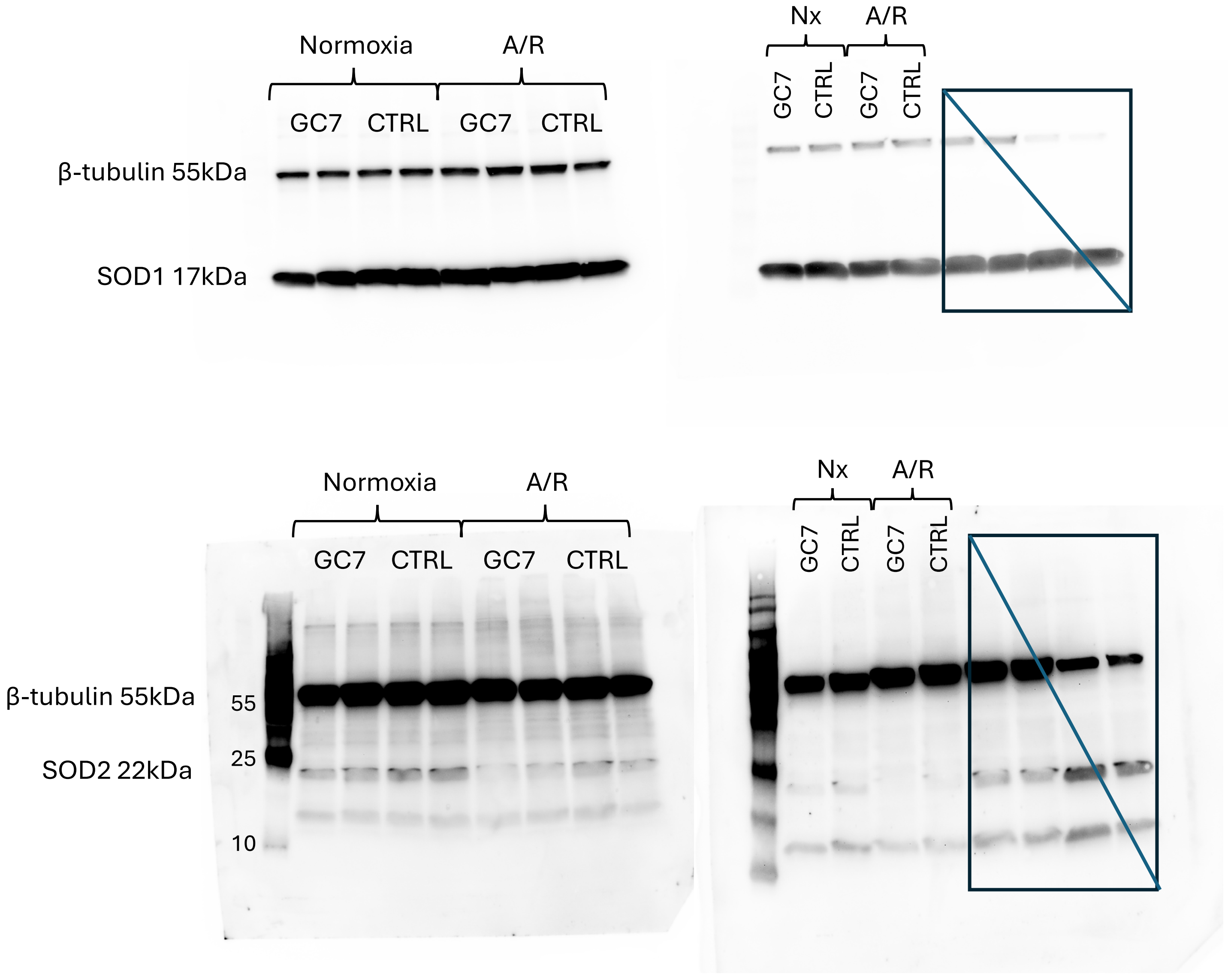

Whole blots for Supplementary Figure 4C

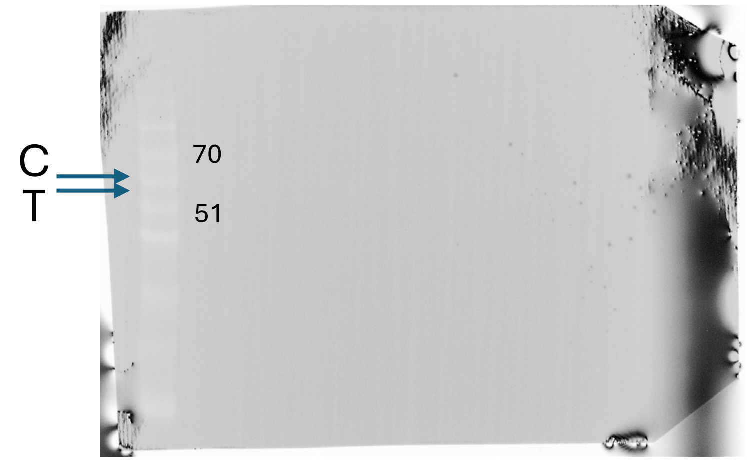

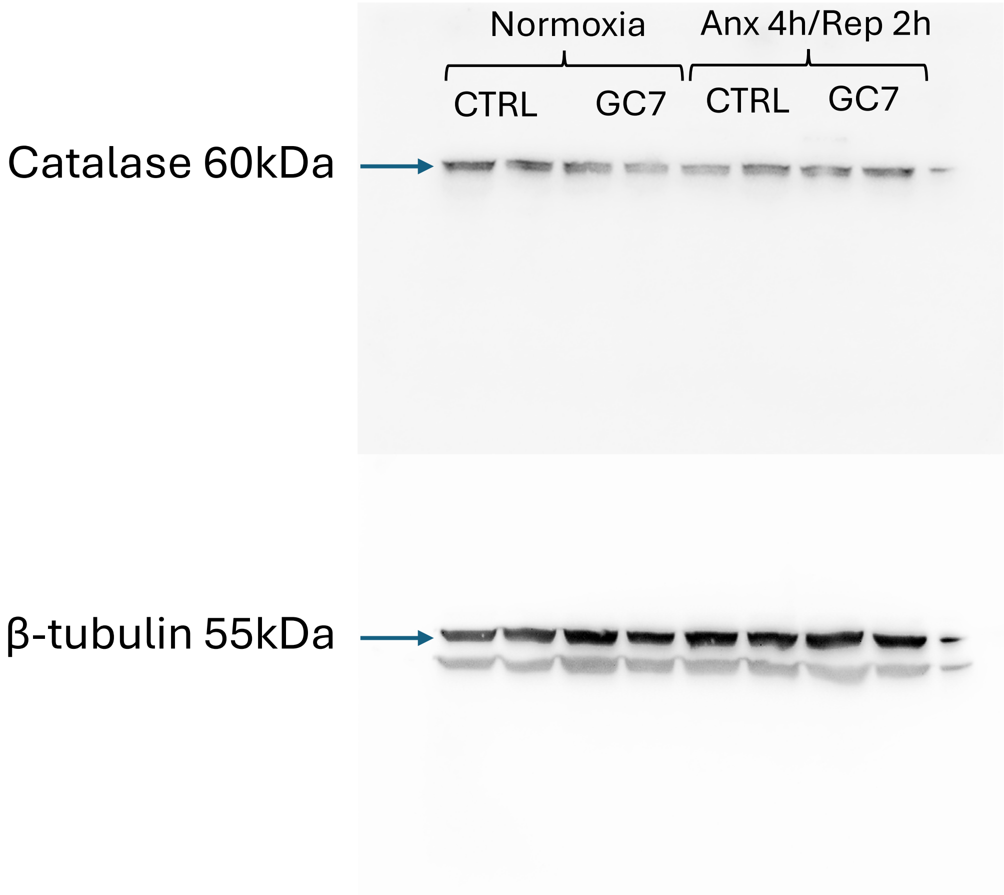

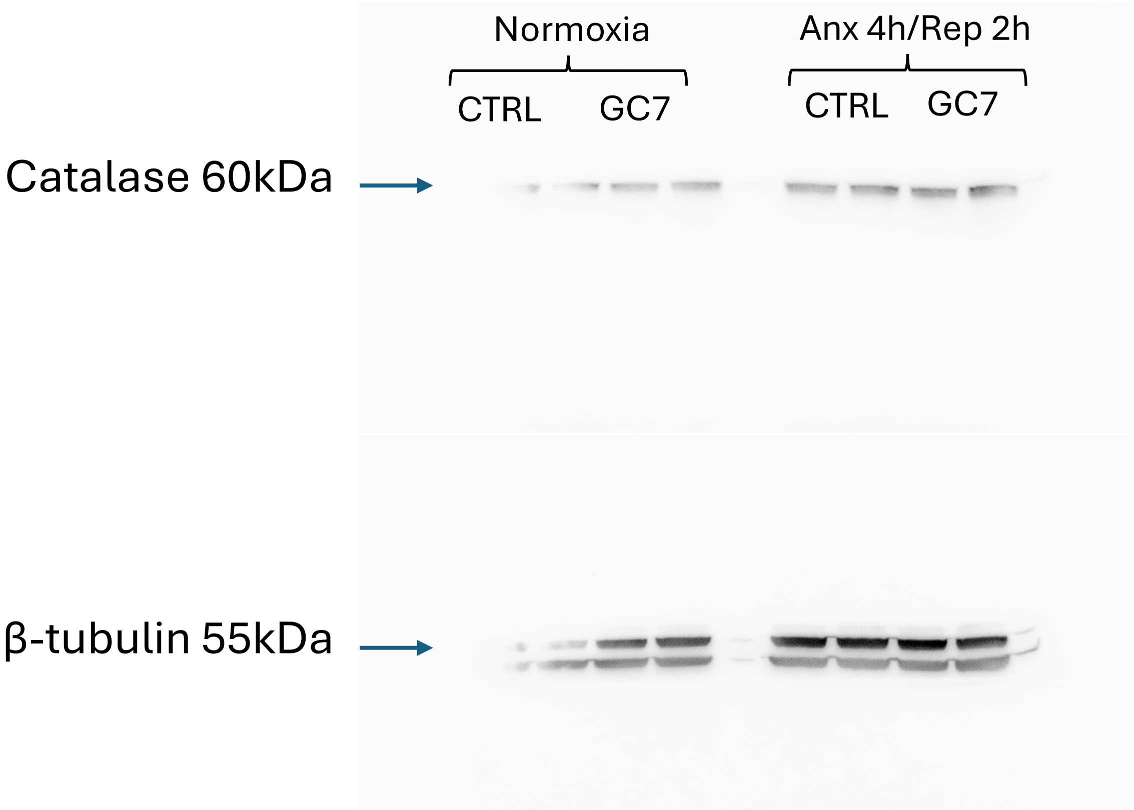

Supplement: Supplemental Figure 5 — Related to Fig. 7. H2O2 reduction was assessed in (A) the presence or (B) the absence of various concentrations of catalase and with or without 30 μM of GC7. Dot plots display individual values and mean ± SEM. n = 4. ∗∗∗p < 0.001, ∗∗∗∗p < 0.0001, (A) One-way ANOVA and Tukey post-hoc test for multiple comparison or (B) Mann-Whitney test. [file mmc5.pdf]
